# Implementation and recognition of novel negatively instructed stimulus-response rules

**DOI:** 10.1007/s00426-025-02212-2

**Published:** 2025-11-29

**Authors:** Alexander W. Baumann, Hannes Ruge

**Affiliations:** Faculty of Psychology, Technische Universität Dresden, Dresden, Germany

## Abstract

Instruction-based learning (IBL) is an essential human skill, enabling flexible and efficient application of novel rules. Previous studies focused on instructions specifying affirmative (or positive) stimulus-response (S-R) rules (i.e., if condition A, then execute action X). Despite their frequent occurrence in our daily lives, negative instructions (i.e., if condition B, then do not execute action X but execute alternative action Y or Z) remain underrepresented in IBL research. In the present study, we conducted three experiments in order to assess the basic behavioral characteristics of this particular instruction type. Experiment 1 revealed reduced implementation efficiency relative to positive instructions as indicated by persistently prolonged response times, suggesting a continued impact of the negated ‘*instruction-related’* S-R (B-X) association. At the same time, a distinct affirmative ‘*implementation-related’* S-R (B-Y or B-Z) association gained strength over the course of repeated rule implementation, as evidenced by a decreasing tendency to alternate between correct response options. This pattern was replicated in Experiment 3. Experiment 2 employed neutral S-R instructions for which type information (positive or negative) was available only during later implementation. The comparison between Experiments 1 and 2 revealed an implementation benefit of advance instruction type information for negative instructions. This suggests that the negation is proactively integrated into the task model for the benefit of upcoming implementation. Overall, our results highlight the unique properties of negative instructions in the context of IBL. In particular, the dual representational nature could enable disambiguation of functional states in rule transformation processes crucial to rapid, novel learning.

**Supplementary Information:**

The online version contains supplementary material available at 10.1007/s00426-025-02212-2.

## Introduction

The ability to learn novel rules via instruction and to apply them at first attempt is an essential and remarkable human skill (Cole et al., 2013). Compared to other types of learning, such instruction-based learning (IBL) happens fast and efficiently, enabling a high degree of cognitive and behavioral flexibility (Cole et al., 2017; Liefooghe et al., 2018; Meiran et al., 2017; Ruge et al., 2018; Wolfensteller & Ruge, 2012). IBL is commonly understood as the rapid learning of novel, often unique, arbitrary rules that are presented prior to actual overt rule execution (Cole et al., 2017; Ruge & Wolfensteller, 2010). According to theoretical accounts of IBL (e.g., Brass et al., 2017), newly instructed stimulus-response (S-R) rules are initially received in a declarative or symbolic format and – critical for IBL success – need to be converted into a procedural, pragmatic or actionable format subsequently. Several attempts to characterize this so-called ‘symbolic-pragmatic transfer’ (Ruge & Wolfensteller, 2010) have been made. Typically, such studies have employed affirmative or ‘positive’ instructions that are conveyed unambiguously by directly specifying the action that needs to be executed in a specific stimulus condition (e.g., Cole et al., 2010; Formica et al., 2020a; Liefooghe et al., 2012; Meiran et al., 2015; Pfeuffer et al., 2018; Ruge et al., 2024). A simple real-world example might be a situation during a basketball game, in which a coach instructs a player to shoot when receiving the ball behind the three-point line in order to gain 3 points (i.e., stimulus A – action X). However, different from this standard form of positive instructions, in other situations the instruction indicates which particular action should be avoided, yet without directly specifying which of several alternative actions to choose instead. To stick with the basketball example, the coach might instruct the player to *not* shoot when receiving the ball directly in front of the three-point line (i.e., stimulus B – [not] action X). In this case, the player can choose among several alternative action options (e.g., dribble the ball [action Y] or pass it to a teammate [action Z]) and execute one of it. This is an example of what has been called ‘negative instruction’ in the past (Geissler, 1912; Langfeld, 1913) – an expression which will also be used in the present paper. Here, a stimulus-response association is accompanied by a negation operator indicating that (i) the specified action is not to be executed and (ii) an alternative action needs to be performed instead. Despite their frequent occurrence in our daily lives and the interesting properties they bear (for a review, see Proctor & Xiong, 2017) such negative instructions are rather neglected in research on rapid instruction-based learning processes. Therefore, the goal of the current study is to characterize rapid novel rule learning after negative instructions. Moreover, we aim to assess the potential relevance of negative instructions and their underlying rule representations for advancing the general understanding of IBL processes.

While little is known about how negative instructions are processed during IBL, certain facets have been examined in a few previous studies. To start with, Liefooghe et al. (2016) found a strong impact of explicitly instructed NoGo rules during the execution of a competing task even when these rules were currently irrelevant and had never been applied before. Hence, they concluded that an effective representation had immediately been formed on basis of the ‘do not’ instruction. Notably, this instruction did not specify an actual S-R association but instead responding was supposed to be inhibited altogether. Crucially, this study and others utilizing NoGo elements (Demanet et al., 2016; Wenke et al., 2009) lack one defining element of negative instructions, namely, to execute an alternative action that is inferred from the originally instructed negated S-R link. This highlights a key difference between NoGo instructions and negative instructions: Despite having the ‘do not’ in common, an action is supposed to be completely withheld in the former but not in the latter.

Another facet of negative instructions is that the actually to-be-implemented S-R rule needs to be inferred from the instructed, negated S-R rule. This aspect was examined in a study in which the authors have focused on the difference between directly instructed and indirectly inferred S-R rules (Pereg & Meiran, 2021). In each experimental trial, a set of two S-R rules was presented but one had to be inferred indirectly from the other, directly instructed rule (e.g., ‘P’ is associated with the right key, the other is ‘T’). As the experimental setting involved two response options, the indirectly instructed (i.e., inferred) rule always involved the alternative key (which would be the left key in the example). It was found that performance on explicitly instructed rules was more efficient than on inferred rules at the very first implementation trial. However, while the study byPereg and Meiran(2021) comprised the element of an indirectly inferred rule, it lacked an explicit negation (or ‘do not’) component.

Broadening the perspective beyond the IBL literature there are several interesting findings on how a negation is processed compared to its affirmative counterpart (Kaup & Dudschig, 2020) – mostly inspired by its important role in language processing (Horn, 1989). For instance, focusing on negation implementation and using lateralized readiness potentials, Dudschig and Kaup (2018) have shown that negation indeed results in initial below-threshold activation of the negated action in motor cortex that is then followed by activation of the factually correct (i.e., alternative) action. This implies competition of two conflicting representations requiring upregulation of control mechanisms (Dudschig & Kaup, 2018). Importantly, different from typical IBL studies, the Dudschig and Kaup setup did not involve the learning of novel S-R instructions. Instead, subjects directly enacted on-screen commands such as ‘now right’ or ‘not right’, likely relying on pre-existing highly overlearned word-direction associations. More closely related to research on instruction-based *learning*, Wirth et al. (2023) used a finger tracking approach and found performance to be adversely affected by negated as compared to affirmative rules due to the tendency to erroneously activate the negated response option. These performance decrements could not even be eliminated by extensive practice (Wirth et al., 2023). In this case, instructions on which (negated) S-R rule to follow were presented before each individual trial, but the rule identities themselves remained the same throughout the entire experiment as a means to study the potential impact of extensive practice. This is different from standard IBL studies which are interested in rapid transformation and learning processes during the initial stage of implementing newly instructed S-R rules (e.g., Formica et al., 2020a; González-García et al., 2020a; Ruge et al., 2019; Ruge & Wolfensteller, 2010).

**Fig. 1:**
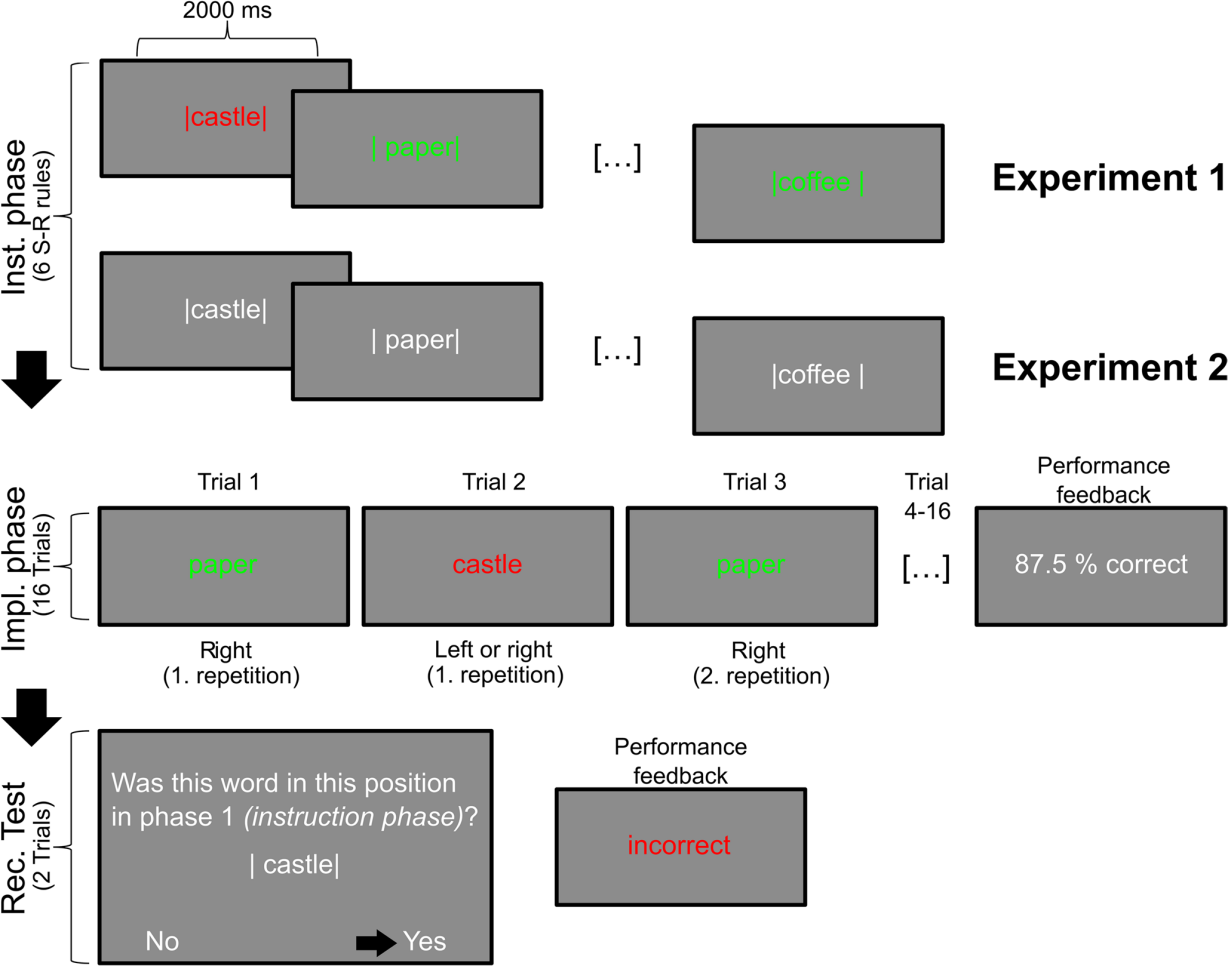
Schematic illustration of one learning block in Experiments 1 and 2. Stimulus-response (S-R) associations were initially introduced during an Instruction (Inst.) phase in which no responses (i.e., key presses) were to be made. A word’s position (and thus an associated response key) was defined by two vertical bars. In Experiment 1, colored instruction presentation allowed for inference on how the S-R associations had to be used subsequently: 3 out of 6 total instructed rules were presented in green (positive: if *paper* press the right button) and the other 3 were presented in red (negative: if *castle* do not press the middle button) whereas in Experiment 2 all rules were presented in neutral white. In the subsequent Implementation (Impl.) phase, two rules of each type had to be executed 4 times by means of button presses. In both Experiments, word stimuli were presented in color: For Experiment 1 the color per word stimulus was the same as in the Instruction phase whereas for Experiment 2 color (bearing the same meaning as in Experiment 1) was introduced only here and remained constant for each stimulus across the entire phase. At the end of each Implementation phase, subjects received performance feedback for the entire phase (87.5% would equal 14 correctly executed rules). Each learning block was concluded by 2 trials of a Recognition (Rec.) Test. Participants were required to indicate whether a word was shown at the same relative position (again defined by the two vertical bars) as in the Inst. phase (here, *castle* was at the middle position during the Instruction phase and at the right position during the Recognition test, thus, requiring a ‘No’-response). During the Recognition Test all rules were presented in neutral white and subjects received performance feedback (i.e., correct or incorrect) for each of the two tests individually

Overall, negative instructions are rarely studied, especially when it comes to rapid learning of novel rules and their initial application. This calls for a reconciliation of findings from negation research (e.g., Kaup & Dudschig, 2020) with empirical (e.g., Baumann et al., 2023; Ruge et al., 2019) and theoretical (Brass et al., 2017) perspectives on IBL in order to broaden the understanding of how instructions are translated into appropriate behavior. To this end, we conducted three experiments employing variations of the same basic paradigm (see Fig. 1 for a schematic illustration).

Each experiment comprised multiple learning blocks that, in turn, were subdivided into three distinct, consecutive phases. In a first instruction phase a set of novel S-R associations had to be encoded without executing any response. Although our primary interest was in characterizing negative instructions, we also included the standard positive instruction type as a reference. In a second implementation phase some of the instructed rules had to be implemented and applied repeatedly. The two instruction type conditions (i.e., positive or negative) were matched with respect to the instruction (i.e., both involved one S-R association) but not necessarily with respect to implementation demands. In the positive instruction condition, correct rule implementation was defined by executing the response that was associated with the stimulus previously (e.g., phase 1: A-X; phase 2: stimulus A requires response X). In the negative condition, correct rule implementation was defined by executing one out of the two response options that had not been associated with the stimulus during the first phase (e.g., phase 1: B-X; phase 2: stimulus B either requires response Y or response Z). In Experiment 1 and Experiment 3, for each S-R association, color-coded instruction type information was already part of the instruction phase enabling type-specific preparatory processes for positive versus negative instructions. By contrast, in Experiment 2, instruction type was revealed only during the implementation phase disabling type-specific preparatory processes. These instructions are therefore labeled ‘neutral’ (see ‘aspect 1’ further below for elaboration). In a final third phase, recognition of the S-R associations presented during the instruction phase was probed. Importantly, instruction type information (i.e., positive or negative) was not part of this test and both, previously implemented and non-implemented associations were tested (for details, see Experiment 1, 'Procedure' section). The latter provided a ‘pure’ estimation of an S-R associations’ representational integrity unbiased by rule implementation.

For conceptual and terminological clarity, throughout this paper, we draw a clear distinction between *instruction-related* S-R representations and *implementation-related* S-R representations. To start with, positive and negative instructions alike are based on word-position associations (see Fig. 1). Since the relative word position implies the eligible response option(s) to be executed during the subsequent implementation phase, a word-position association also exhibits features of a word-response association. In case of positive instructions, the identities of word-position associations and eventually implemented word-response associations are identical. Hence, the term word-response (i.e., stimulus-response) association would be unambiguous and intuitively appealing. However, due to the more complicated situation in case of negative instructions, terminology needs to be chosen more carefully. According to our working hypothesis based on previous negation research, we assume that following a novel negative instruction an *instruction-related* representation is formed in a first step. By this, we refer to the negated S-R association (i.e., B-X) to which the negation operator (i.e., *not*) is attached. This association is probed in the recognition test. In a second step, an *implementation-related* representation is formed that comprises an alternative, affirmative S-R association derived from the initial instruction-related representation (i.e., either B-Y or B-Z). In other words, the very same stimulus is associated with two responses of which one refers to the instructed, negated response and one refers to the actually implemented response(s). Thus, the interplay of instruction-related S-R association and implementation-related S-R association(s) following negative instructions might open a window into the dynamics of response conflict in IBL (cf. Baumann et al., 2023).

The overarching aim of this study was to assess behavioral characteristics following novel, negative instructions while specifically focusing on the relative contribution of instruction-related and implementation-related S-R associations and their interplay. For that purpose, we specified three core aspects for which we detail our considerations and hypotheses below.

### Proactive instruction-induced rule transformation processes prior to implementation

We assumed that behavioral performance following negative instructions would lie within a spectrum bounded by the effects of positive instructions on the one hand and those of ‘neutral’ instructions on the other hand. Positive instructions, at the upper end of this spectrum, enable maximally efficient instruction implementation. As previous research suggests, this is mainly due to ’proceduralization’ (Brass et al., 2017) prior to the first implementation trial. That is, declarative representations are proactively transformed into an actionable, procedural state – most likely facilitated by motor imagery or covert practice of the instructed S-R rule in the absence of overt responding (Fregni et al., 2024; Liefooghe et al., 2021; Palenciano et al., 2024; Ruge & Wolfensteller, 2010; Theeuwes et al., 2018). In contrast, and at the lower end of the spectrum, ‘neutral’ S-R instructions should not reasonably allow for any such proactive transformation into an actionable procedural state. The reason is that neutrally instructed S-R associations are only affirmed or negated during the upcoming implementation phase which effectively eliminates the possibility to prepare any response. Hence, for neutral instructions we expected maximally inefficient instruction implementation due to the lack of proactive transformation processes.

For negative instructions – and relative to the positive and neutral instruction reference points – two, quite different scenarios can be envisioned. According to the first scenario reminiscent of so-called ‘fusion models’ of language (Horn, 1989; Lyons, 1995), the instruction-related, negated S-R association is proactively re-coded into an affirmative implementation-related S-R association. In the extreme case, the original, might even be entirely replaced and subsequent implementation would hence be equivalent to the implementation of positive instructions. Accordingly, no performance cost would be expected for negative relative to positive instruction implementation.

This contrasts with a second scenario, in which rule transformation – in the sense that an actionable S-R association is formed and can be readily applied – might not at all take place proactively prior to actual rule implementation. Hence, this transformation would be completed reactively only at the first encounter with an actual implementation trial. Consequently, in the extreme case, only the instruction-related S-R association would be encoded initially during the instruction phase, that is, only the *not* to be executed action would be linked to the stimulus. An actionable implementation-related rule would be derived reactively once actual implementation is required in the first implementation trial. In turn, initial implementation performance should be slowed down relative to positive instructions while being at a similar level as following neutral instructions.

### Evolution of novel rule implementation during early practice

If negative instructions did entail initial implementation cost for reasons explained under point 1 above, the next crucial question would be whether this cost persists across repeated implementation trials or whether performance would gradually approach a similar level as following positive instructions. Despite the limited effect of extensive practice on well-practiced negated rules (Dudschig & Kaup, 2018; Wirth et al., 2023), we tentatively hypothesized an incremental relative efficiency improvement in the negative condition when specifically zooming into the first few implementation trials. This expectation is derived from earlier findings of steeper efficiency increases in more difficult (here: negative vs. positive) instruction conditions during repeated rule execution (Baumann et al., 2023; Ruge et al., 2019).

In this context, and unique to the negative instruction condition, the experimental setup involving three response options allowed us to utilize response switching behavior across multiple implementation instances of the same instructed S-R rule as a valuable additional performance indicator. The reasoning goes as follows. On the one hand, if rule implementation was fully determined by the instruction-related S-R association, this association (e.g., B-X) would be retrieved, the negation operator would be applied and both alternative (and correct) response options (Y and Z) would be activated with a roughly similar probability at a sub-threshold level. Consequently, from one stimulus repetition (e.g., Stimulus B, Repetition 1) to the next (e.g., Stimulus B, Repetition 2) a high tendency to switch between response options could be expected (i.e., Response Y at Repetition 1 and Response Z at Repetition 2). On the other hand, if a distinct implementation-related representation was established at a given trial (e.g., Stimulus B, Response Z), the chosen response option Z should be activated with a higher probability on the next repetition of the same stimulus B. This, in turn should be resulting in reduced switching tendency (i.e., repeated execution of Z given B). Thus, a higher switching rate indicates a stronger reliance on the instruction-related S-R association and, conversely, a lower switching rate indicates the reliance on one of the two eligible implementation-related S-R associations. Based on this line of reasoning, a switching rate decrease across the implementation phase would indicate increasing reliance on one distinct implementation-related S-R association. This emphasizes the informational value of the switching measure in tracking (representational) stability over the course of repeated rule application. Importantly, however, this logic does not apply to the positive instruction condition, as, here, a response switch is always associated with inappropriate rule implementation. Nevertheless, switching rate in the positive condition – when considering switches between both alternative (and here: incorrect) response options – can be used as an empirical baseline measure of a general, seemingly random tendency to alternate between response options in the absence of trial-specific feedback.

### Integrity of the instruction-related S-R association

For negative instructions, the recognition test is especially informative with respect to the interplay of instruction-related and implementation-related S-R associations. On the one hand, the instruction-related (i.e., negated) S-R association might be overwritten by the implementation-related (i.e., alternative affirmative) S-R association. This might either happen immediately already during the instruction phase or over the course of several implementation trials (cf. Monsell & Graham, 2021). In the extreme case, the memory trace of the original instruction would become inaccessible resulting in chance-level performance. On the other hand, the instruction-related S-R association might be maintained over the course of the entire implementation phase. The preserved integrity of the instruction-related S-R association might benefit from the presence of non-binary response options (Orenes et al., 2014) and from subjects retrieving the original association on every implementation trial in order to newly derive one of the two alternative response options for implementation (see considerations on response switching above). In this case, accuracy rates close or even equal to positive instructions – for which no potential conflict between implementation and recognition exists – would be expected. Taking both of these extremes into account, we hypothesize that, following implementation, recognition test accuracy rates for negatively instructed rules should be well above chance-level but lower than for positively instructed rules.

At last, we explored the correlation between these estimates of instruction-related S-R association integrity (i.e., recognition test accuracy) and implementation markers (i.e., implementation efficiency and stability). We reasoned that a potential relationship between these behavioral indices would be informative regarding the extent to which rule implementation would be impacted by instruction-related S-R association representation.

## Experiment 1

In Experiment 1 we introduced negative instructions by adapting an established IBL-paradigm (Ruge et al., 2019). To ensure that recognition test performance was not heavily influenced by task commitment (or a lack thereof) we decided to relate rule implementation and rule recognition to each other by means of reward (for details, see below).

The explicit aim of Experiment 1 was to characterize early implementation behavior after novel negative instructions relative to standard positive instructions in rapid IBL. In particular, we were interested in the relative contribution of instruction-related and implementation-related S-R associations.

### Methods

#### Participants

A total of 33 participants (mean age = 24.3, SD age = 5.36; 26 females and 7 males) were recruited from the subject pool of Technische Universität Dresden. All participants were native German speakers, had normal or corrected-to-normal vision and reported absence of congenital red-green color blindness. Participants gave written informed consent in accordance with the Declaration of Helsinki before the start of the experiment and received 10 Euro or were compensated with course credit for their participation. Depending on their performance in the experiment, subjects could gain up to 5 Euro of additional payment (mean = 3.20€; range: 1.50€ to 4.30€). This study was not preregistered. All experimental procedures followed the ethical guidelines of the German Psychological Society. According to the regulations of the German Research Foundation (Deutsche Forschungsgemeinschaft) that funded our research, experiments like those described in this study do not require formal approval from a research ethics committee. This is because the study was associated with neither deception nor a risk of physical or emotional harm and did not involve clinical intervention or examination and also did not involve underaged, or elderly participants (see http://www.dfg.de/foerderung/faq/geistes_sozialwissenschaften/index.html).

Based on implementation phase performance, 3 subjects were excluded from the analyses: Two subjects performed below chance level in the negative condition and for one subject unallowed keypresses were registered on more than 20% of all rules during the instruction phase. Therefore, the final sample included 30 subjects.

#### Task and materials

The experiment comprised 36 learning blocks each subdivided into 3 phases.

In the first phase (the instruction phase) participants were presented with 6 novel written words (disyllabic German nouns) which were framed by two vertical bars and which were displayed centrally before a grey background on a computer screen. The position of a noun relative to the vertical bars defined the response to be implemented later on. The noun could be positioned in 3 different ways relative to the vertical bars: exactly in the middle between them, closer to the left one or closer to the right one. These relative positions corresponded to the middle, the index, and the ring finger of the right hand, respectively. Noun and vertical bars were displayed either in red or in green color denoting negative vs. positive instructions. The correct response to a given noun was determined by the combination of its color and position: Green (i.e., positive) instructions indicated a response that is spatially congruent to the noun’s position (e.g., if the noun is closer to the right bar, a right – ring finger – response is correct). By contrast red (i.e., negative) instructions indicated a spatially incongruent response (e.g., if the noun was closer to the right bar, a right – ring finger – response is incorrect, but a middle as well as an index finger response would both be correct). Participants were explicitly informed about the meaning of both colors in the study context (for this general instruction, see Supplementary Materials, Sect. 1 and Supplementary Fig. 1) and were told not to make any response in the instruction phase.

In the second phase (the implementation phase) 4 out of the 6 nouns from the instruction phase reappeared on screen 4 times each. This time, the nouns were displayed without the vertical bars, again centrally, in the same color as during the instruction phase. Participants were now required to execute the response according to the previous instruction. In order to avoid interference with trial-and-error learning during implementation, subjects did not receive feedback after individual implementation trials but only at the end of each implementation phase.

The third phase within each of the 36 learning blocks comprised two trials of a recognition test. In each trial subjects were presented with one of the previously instructed nouns again framed by two vertical bars, but this time in white color. Subjects had to indicate whether or not the noun appeared in the same relative position as in the instruction phase. The aim of this recognition test was to specifically probe memory for the *instruction-related* S-R association (i.e., in case of negative instructions, the negated S-R association detached from negation operator).

Importantly, both aspects of IBL probed in this experiment – actual rule implementation and rule recognition – were connected via reward. Per learning block, subjects received one point for each correctly implemented rule in the implementation phase (maximum = 16 points). The number of points earned was presented to the subjects alongside performance feedback at the end of the implementation phase. The total number of points per learning block was now determined by recognition test performance: if both recognition test responses were incorrect, implementation phase points were divided by 2; if one response was correct, implementation phase points remained unchanged; if both responses were correct, implementation phase points were doubled. The reasoning behind this rewarding policy was to strengthen the relevance of the recognition test without diminishing the importance of implementation phase performance.

A QWERTZ-keyboard was used for responding with the keys T, Z and U corresponding to the stimulus positions. Participants were explicitly asked to place the index, middle and ring finger of their right hand on top of these keys and to only use them for responding during the implementation phase. Undesired keypresses during the instruction phase were registered and subjects were reminded to not yet respond. Per participant, the nouns were randomly assigned to learning block, instruction type and relative position. For the recognition test a different set of response keys (left and right arrow keys) had to be used to indicate whether or not the current stimulus position matched the originally instructed one. This was done to minimize possible response congruency and interference effects between the implementation tests and recognition test. The experiment ran on PsychToolbox Version 3.0.15 under Matlab 2019b.

#### Procedure

All text displayed during the experiment was in German. All warning texts were displayed in blue color. Each phase per learning block was preceded by a phase-specific announcement (Phase 1: Memorize; Phase 2: Execute; Phase 3: Recognition Test) being displayed centrally on screen in white color for 2000 milliseconds (ms).

Following the instruction phase announcement, each of the 6 instructed S-R associations was displayed for 2000ms. If a key was pressed during the instruction phase the current S-R rule display was replaced by a reminder to not press a key which remained on screen until the next S-R association appeared. At the end of each instruction phase, a fixation cross was displayed for 2000ms. In each instruction phase, there was one S-R association per instruction type and position (i.e., left, middle, right). This resulted in 3 positive and 3 negative instructions per instruction phase, with each response option being linked to a stimulus of each instruction type once. The order in which the instructed S-R associations appeared on screen was randomly determined in each learning block.

The implementation phase announcement was followed by a fixation cross being displayed for 1000ms. Subsequently, each stimulus was presented for a maximum duration of 2000ms or until a subject had executed a response. If no response was executed after 2000ms the words ‘too slow’ were displayed for 500ms. Similarly, if a key outside the predefined set of response keys was pressed, participants were notified by the message ‘Wrong key: Respond with keys T, Z or U’ being displayed for 1000ms. Four nouns (out of six) from the instruction phase were repeated 4 times each resulting in a total of 16 trials per implementation phase. Nouns were presented in pseudorandom order such that direct stimulus repetitions were avoided. Two of the 4 to be implemented rules were pseudo-randomly drawn from each instruction type such that each of the 3 instructed responses was involved at least once. Subjects did not know beforehand which of the words would re-appear in the implementation phase. Note, that in the implementation phase all word stimuli were presented in the same color as in the instruction phase (i.e., either green or red for positive or negative instructions, respectively). At the end of each implementation phase subjects received feedback regarding their performance. The percentage of correct responses was displayed on screen for 2000ms.

As the last part of each learning block the recognition test started after implementation phase performance feedback. Two recognition test trials followed: One out of the 6 nouns from the instruction phase of the very same learning block – this time again framed by two vertical bars – was displayed centrally on screen. The noun could either be in the same or in a different position with respect to the instruction phase. The explicit question whether this word had appeared in the same position in the instruction phase was displayed in the top half of the screen. The response options ‘no’ and ‘yes’ were displayed in the bottom left and bottom right corner of the screen, respectively. All these elements remained on screen for 10s or until a key was pressed. For the recognition test the left and right arrow keys had to be used for responding. Subjects received immediate feedback regarding their recognition test performance via the words ‘correct’ or ‘incorrect’ being displayed centrally on screen in green or red color, respectively. If a key other than either the left or the right arrow key was pressed subjects were notified that they had pressed a wrong key. If no response at all was provided subjects were told to respond faster. Both messages were accompanied by the explicit reminder to use the arrow keys for responding. All feedback texts remained on screen for 1000ms. After this performance feedback the second recognition test trial started immediately. Importantly, in one recognition test trial one of the four rules that were carried over into the implementation phase (i.e., previously implemented) was probed whereas one of the remaining two rules (i.e., merely instructed and not implemented) was probed in the other recognition test trial. The order of the two recognition tests (i.e., on previously implemented or not implemented rules) was random. For the recognition test on not implemented instructions, selection of the to be tested S-R association was random. For the recognition test on implemented instructions, rules were pseudo-randomly selected such that a total of 18 rules per instruction type were probed over the course of all 36 learning blocks. This randomization scheme permitted that by chance both recognition test trials could be associated with the same instruction type. In half of the recognition test trials, the probe matched the S-R association presented during the instruction. Thus, different from the implementation phase, the chance level was at 50% for both instruction type conditions in the recognition test.

Individual performance feedback for the second recognition test was followed by a fixation cross being displayed for 1000 ms. Then, subjects were informed about the total amount of learning block points. The message ‘You earned X points in this learning block’ was displayed centrally on screen for 2000ms. At the very end of the experiment the total points from the individual learning blocks were summed up and divided by the maximum number of points (i.e., 36*16*2 = 1152). The multiplication of the resulting fraction with 5 then determined the final additional payout to subjects.

Before the start of the actual experiment, the experimental task was practiced in two training blocks. If – in the implementation phase – subjects pressed a key less than 200ms after stimulus onset they were notified about their premature reaction and reminded to only react once the stimulus appeared on screen. This warning remained on screen for 1000ms. It was exclusively used during training to sensitize subjects for responding only after the stimulus had actually been visible and processed – no such warning was displayed in the actual experiment. Each noun was used in one learning block only and no noun from training reappeared in the actual experiment.

#### Analyses

Analyses were performed on the dependent variables Accuracy (i.e., proportion of correct trials), Response Time (RT) and Response Switches (i.e., proportion of trials in which the response to the current trial did not match the response of the previous trial). Trials in which a response was executed within the first 200ms after stimulus onset as well as response omission trials were excluded from the analyses. Only correct trials were considered in the RT analyses. By means of repeated measures analysis of variance (ANOVA) we examined the hypothesized impact of the *instruction type* (positive vs. negative) and *stimulus repetition* (1 to 4). Greenhouse-Geisser correction was applied where necessary and follow-up t-tests were corrected for the number of tests using Bonferroni correction.

It is important to mention, that due to the experimental setup encompassing 3 response options there is a natural imbalance regarding the chance level with which a correct response could be expected. While exactly one response is correct in the positive instruction condition resulting in a chance level of 33.3%, the chance level is at 66.7% in the negative instruction condition due to two correct response options per instruction. Thus, while implementation accuracy results will be reported, they do not constitute a major focus in this study. However, in terms of the recognition test – where the chance level is at 50% in both instruction conditions – we considered recognition accuracy the most important indicator of S-R association integrity with respect to both the comparison between instruction conditions and when exploring the relationship between implementation and recognition test performance.

Response switching rate was calculated as the proportion of trials in which the executed response changed from the previous to the current stimulus repetition. Each response switch can be categorized as one of three types: From the instruction-related to an alternative response (instructed-to-alternative), from an alternative to the instruction-related response (alternative-to-instructed), and from one alternative to the other alternative response (alternative-to-alternative). While the first two switch types are always associated with one error^1^, the interpretation of the last is strongly dependent on the instruction context: Importantly, in the negative condition this represents switching between two equally correct options while in the positive condition both alternative options are incorrect. In order to capture this aspect of switching behavior, *switch type* was included into the analyses on switching behavior as an additional factor. Here, we expected that alternative-to-alternative switches in particular would drive potential differences between conditions.

This switching definition provides a holistic overview over all kinds of response switching by taking into account all trials (or, more precisely, all consecutive stimulus repetition sequences). Alternatively, switching behavior in the negative condition could also be examined by taking into account the proportion of (alternative-to-alternative) switches amongst all consecutively correct stimulus repetitions. Naturally, this kind of switching rate can only be calculated for the negative instruction type (as for positive instructions, a switch can never be associated two consecutively correct stimulus repetitions). This alternative definition was applied in a complementary analysis by means of a one-way repeated measures ANOVA (see Supplementary Materials, Sect. 2 and Supplementary Fig. 2).

To statistically evaluate the recognition test results, a repeated measures ANOVA on Recognition Accuracy was conducted including the two within-subjects factors *instruction type* and *implementation status* (implemented vs. not implemented). Exploratory analyses focusing on the relationship between implementation phase performance and recognition test performance were conducted. The Pearson correlation coefficient was used as the measure of association strength. In order to identify possible trends across repetition instances, correlation coefficients were computed separately for each stimulus repetition of the implementation phase. Analyses were carried out with IBM SPSS 28 and Matlab 2018b. Subjects’ raw behavioral data are available at https://osf.io/839dk/. Further experimental materials can be made available upon request.

### Results

#### Implementation: accuracy

**Fig. 2:**
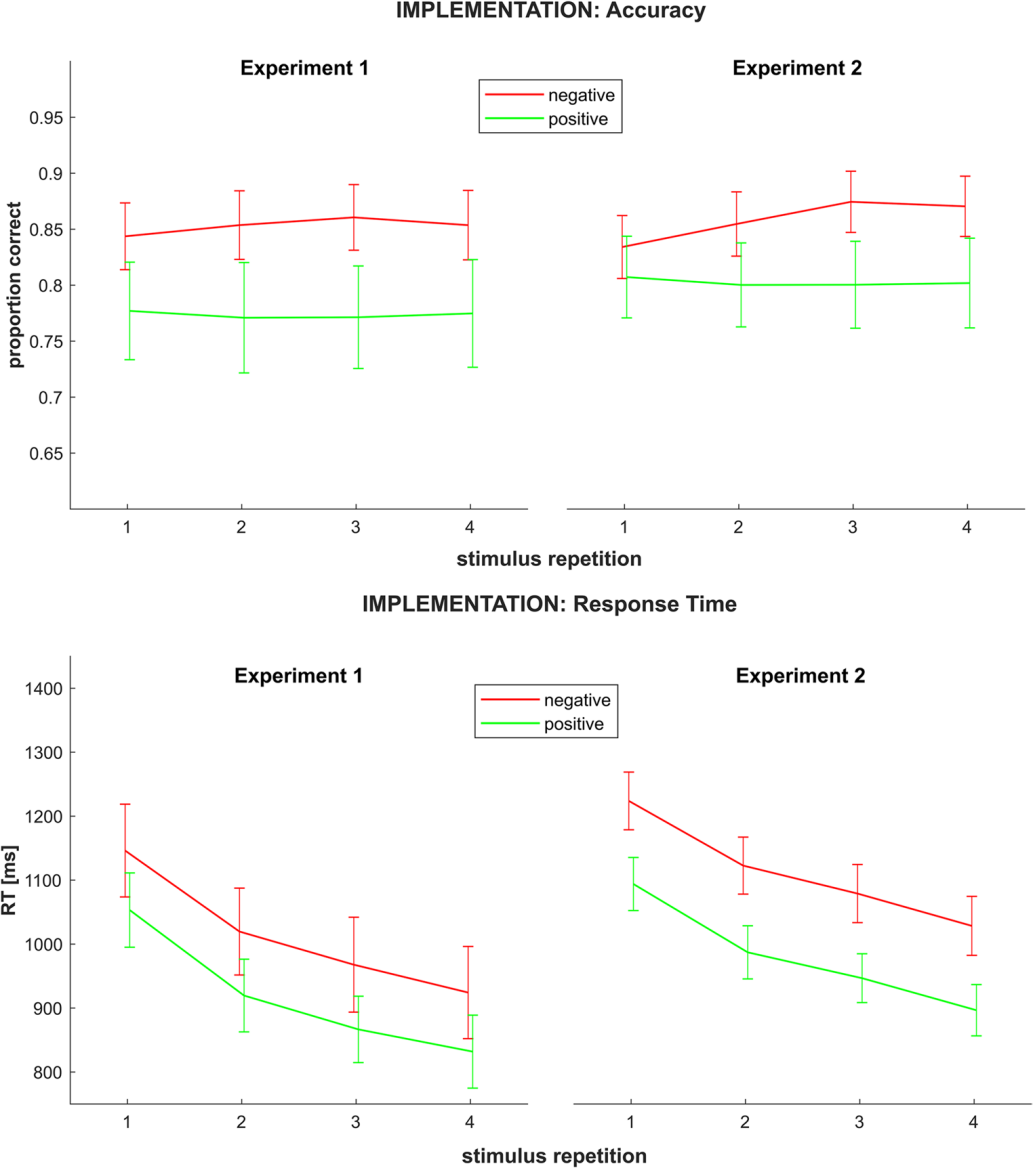
Implementation phase accuracy (upper panel) and RT (lower panel) by rule type and stimulus repetition in both experiments. Error bars represent 95% confidence intervals

A significant main effect of *instruction type* (F_1,29_ = 19.53; *p(F)* < 0.001; *η*_*p*_^*2*^ = 0.40) indicated higher accuracy rates for negatively than for positively instructed rules. As mentioned above, this most likely trivially reflected the chance level imbalance between the two conditions. Neither the main effect of *stimulus repetition* (F_3,87_ = 0.27; *p(F)* = 0.845; *η*_*p*_^*2*^ = 0.01, linear contrast: F_1,29_ = 0.38; *p(F)* = 0.545; *η*_*p*_^*2*^ = 0.01) nor the interaction of *instruction type x stimulus repetition* (F_1,29_ = 1.10; *p(F)* = 0.353; *η*_*p*_^*2*^ = 0.04, linear contrast: F_1,29_ = 1.13; *p(F)* = 0.297; *η*_*p*_^*2*^ = 0.04) reached statistical significance. For an illustration, see Fig. 2 (upper panel).

#### Implementation: response time

Significant main effects of *instruction type* (F_1,29_ = 57.92; *p(F)* < 0.001; *η*_*p*_^*2*^ = 0.67) as well as *stimulus repetition* (F_1.48,43.12_ = 150.38; *p(F)* < 0.001; *η*_*p*_^*2*^ = 0.84, linear contrast: F_1,29_ = 182.96; *p(F)* < 0.001; *η*_*p*_^*2*^ = 0.86) were observed. Consistent with our original hypotheses regarding RTs, this indicated longer overall RTs on negatively instructed than on positively instructed rules and generally decreasing RTs from the first to the fourth repetition, respectively. However, contrary to our working hypothesis, no significant interaction effect of *instruction type* x *stimulus repetition* was detected (F_3,87_ = 0.29; *p(F)* = 0.836; *η*_*p*_^*2*^ = 0.10, linear contrast: F_1,29_ < 0.01; *p(F)* = 0.985; *η*_*p*_^*2*^ < 0.01). For an illustration, see Fig. 2 (lower panel). Notably, a follow-up analysis on general task practice effects (that is, performance in the first versus the second half of all learning blocks) showed that this results pattern remained stable throughout the experiment. For a detailed report, please see Supplementary Materials, Sect. 3.

#### Implementation: response switches

**Fig. 3:**
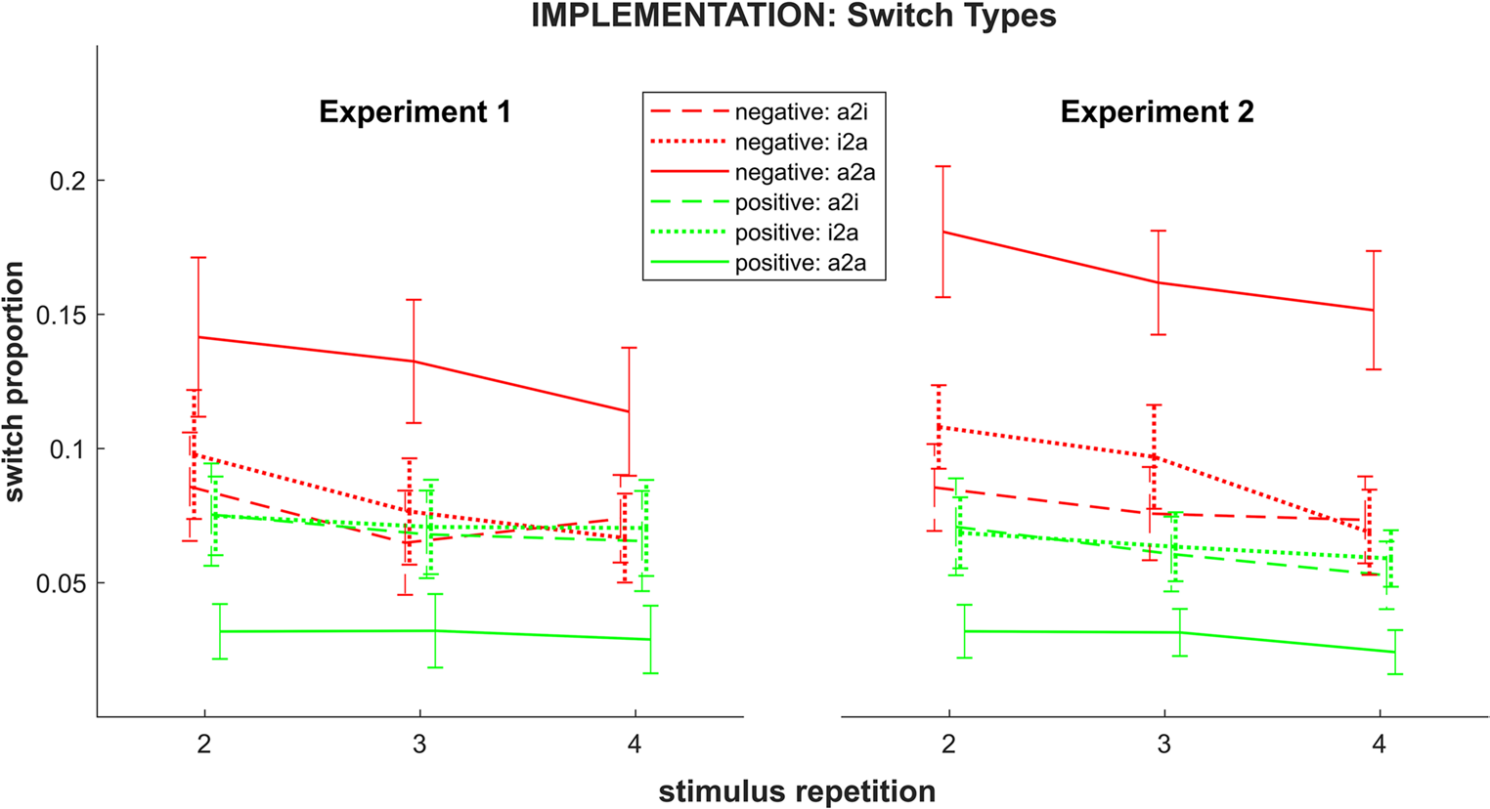
Switching rates in Experiments 1 and 2 by rule type, stimulus repetition and switch type (a2i = alternative-to-instructed; i2a = instructed-to-alternative; a2a = alternative-to-alternative). Error bars represent 95% confidence intervals

Please note, that due to the definition of a response switch only the last three stimulus repetitions could be included into the switch-related analyses. The 2-by-3-by-3 repeated measures ANOVA on response switches (for an illustration, see Fig. 3) revealed significant main effects of *instruction type* (F_1,29_ = 32.40; *p(F)* < 0.001; *η*_*p*_^*2*^ = 0.53) as well as *stimulus repetition* (F_2,58_ = 11.36; *p(F)* < 0.001; *η*_*p*_^*2*^ = 0.28, linear contrast: F_1,29_ = 17.31; *p(F)* < 0.001; *η*_*p*_^*2*^ = 0.37). In line with our hypothesis, this indicated a greater overall tendency to alternate between response options in the negative than in the positive condition and an overall decrease of this tendency across stimulus repetitions.

While there was no significant main effect of the factor *switch type* (F_1.29,37.48_ = 1.40; *p(F)* = 0.253; *η*_*p*_^*2*^ = 0.05), we observed a significant interaction *instruction type x switch type* (F_1.34,38.93_ = 60.86; *p(F)* < 0.001; *η*_*p*_^*2*^ = 0.67). This interaction can be characterized best by paired t-tests: The proportion of alternative-to-alternative switches was significantly higher for the negative than for the positive instruction type (t = 9.32, *p* < .001), whereas neither instructed-to-alternative (t(29) = 1.20, *p(t)* = 0.241) nor alternative-to-instructed (t(29) = 0.51, *p(t)* = 0.612) switching rates differed significantly between instruction types. Furthermore, within the negative instruction type, alternative-to-alternative (here: correct-to-correct) switches were significantly more frequent compared to both, instructed-to-alternative (t(29) = −5.06, *p(t)* < 0.001) and alternative-to-instructed (t(29) = 5.85, *p(t)* < 0.001) switches, whereas the latter – error-related – ones did not differ significantly from each other (t(29) = 1.51, *p(t)* = 0.425). Within the positive condition, alternative-to-alternative (here: error-to-error) switches were significantly less frequent than both other switch types (t(29) = −7.98, *p(t)* < 0.001 and t(29) = −10.51, *p(t)* < 0.001) – with these, again, not differing from each other (t(29) = 0.74, *p(t)* > 0.999). This suggests, that the overall higher switching rate in the negative instruction condition was, indeed, driven by alternative-to-alternative switches.

Crucially, and again in line with our hypotheses, the significant interaction of *instruction type* x *stimulus repetition* (F_2,58_ = 5.59; *p(F)* = 0.006; *η*_*p*_^*2*^ = 0.16, linear contrast: F_1,29_ = 8.12; *p(F)* = 0.008; *η*_*p*_^*2*^ = 0.22) revealed a more pronounced switching decrease in the negative than in the positive instruction condition. To better characterize the interaction effect, we conducted a one-way repeated measures ANOVA per *instruction type* condition. In the positive condition, no significant effect of the factor *stimulus repetition* (F_1.40,40.58_ = 1.56; *p(F)* = 0.223; *η*_*p*_^*2*^ = 0.05, linear contrast: F_1,29_ = 2.18; *p(F)* = 0.151; *η*_*p*_^*2*^ = 0.07) was observed. In contrast, this effect was significant in the negative condition (F_2,58_ = 12.14; *p(F)* < 0.001; *η*_*p*_^*2*^ = 0.30, linear contrast: F_1,29_ = 19.07; *p(F)* < 0.001; *η*_*p*_^*2*^ = 0.40), indicating a strong linear decrease across repetitions. Finally, paired t-tests revealed that switching rate in the negative condition was significantly higher than in the positive condition at all repetition instances (all t(29) > 4.64, all *p(t)* < 0.001).

At last, neither the interaction *switch type x stimulus repetition* (F_4,116_ = 0.94; *p(F)* = 0.442; *η*_*p*_^*2*^ = 0.03, linear contrast: F_1,29_ = 0.37; *p(F)* = 0.546; *η*_*p*_^*2*^ = 0.01) nor the three-way interaction *instruction type x switch type x stimulus repetition* (F_4,116_ = 0.50; *p(F)* = 0.737; *η*_*p*_^*2*^ = 0.02, linear contrast: F_1,29_ = 0.05; *p(F)* = 0.83; *η*_*p*_^*2*^ < 0.01) reached significance.

##### Alternative-to-alternative response switches

As mentioned above, alternative-to-alternative switches are qualitatively distinct in that the outcome (i.e., correct or incorrect) does not change with switching which makes them particularly relevant in the present context. Therefore – despite the absence of a significant three-way interaction – we conducted an additional repeated measures ANOVA focusing exclusively on this switch type.

As expected, significant main effects of *instruction type* (F_1,29_ = 86.88, *p(F)* < 0.001, *η*_*p*_^*2*^ = 0.75) and *stimulus repetition* (F_2,58_ = 4.46, *p(F)* = 0.016, *η*_*p*_^*2*^ = 0.13, linear contrast: F_1,29_ = 8.75, *p(F)* = 0.006, *η*_*p*_^*2*^ = 0.23) indicated a higher overall switching rate in the negative than in the positive condition and a general decrease across the implementation phase, respectively.

Importantly, although the interaction effect *instruction type x stimulus repetition* (F_1.66,47.99_ = 2.30, *p(F)* = 0.120, *η*_*p*_^*2*^ = 0.07) did not reach significance, a significant linear contrast indicated a more pronounced switching rate decrease across repetitions in the negative as compared to the positive condition (F_1,29_ = 4.49, *p(F)* = 0.043, *η*_*p*_^*2*^ = 0.13). Consistent with this pattern, the relative proportion of switches in consecutively correct repetition instances decreased across the implementation phase in the negative condition (see Supplementary Materials, Sect. 2). Conversely, this implies an increase in non-switch proportion in such correct-to-correct sequences.

#### Recognition test: accuracy

**Fig. 4:**
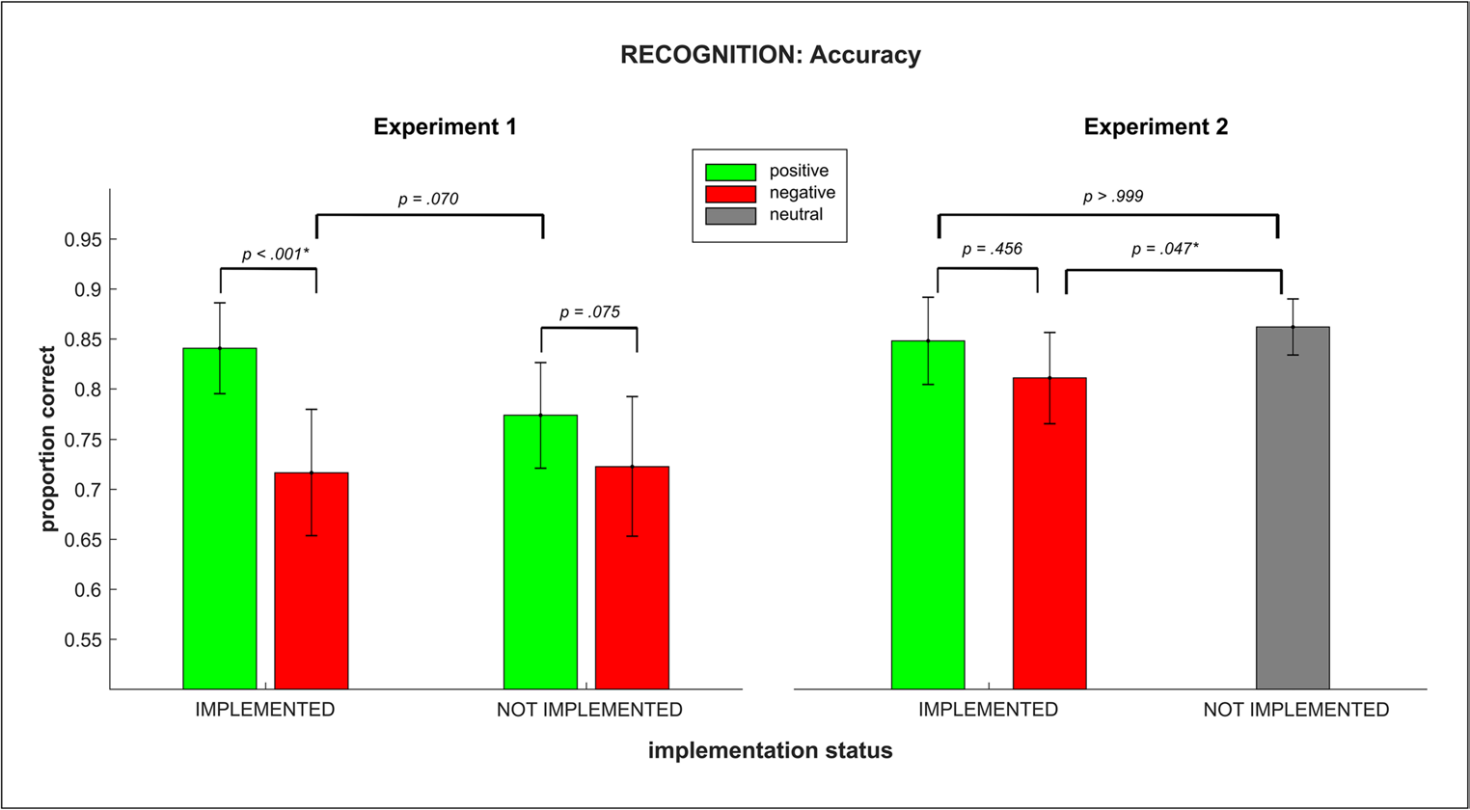
Recognition test accuracy in experiments 1 and 2 by rule type and implementation status. Error bars represent 95% confidence intervals. Asterisks indicate significant pairwise comparisons at *p* <.05

Recognition test accuracy (for an illustration, see Fig. 4) was significantly above chance level in all conditions (all t(29) > 6.27, all *p* < .001). Accuracy rates in the recognition test were compared between conditions by means of a 2-by-2 repeated measures ANOVA. A significant main effect of *instruction type* (F_1,29_ = 16.99; *p(F)* < 0.001; *η*_*p*_^*2*^ = 0.37) indicated generally higher recognition accuracy on positively instructed rules than on negatively instructed ones. Although overall recognition test accuracies on previously implemented rules were numerically higher than those on not implemented rules, the main effect of *implementation status* was only marginally significant (F_1,29_ = 3.55; *p(F)* = 0.070; *η*_*p*_^*2*^ = 0.11). Furthermore, the significant interaction of *instruction type* x *implementation status* (F_1,29_ = 4.77; *p(F)* = 0.037; *η*_*p*_^*2*^ = 0.14) indicated a more pronounced difference between previously implemented and not implemented rules in positive as compared to the negative instruction condition. This can be further illustrated by means of paired t-tests: For the positive instruction type, recognition test accuracies on previously implemented rules were significantly higher than on not implemented rules (t(29) = 3.32, *p(t)* = 0.002). For the negative instruction type, no such difference could be observed (t(29) = − 0.24, *p(t)* = 0.815).

We also compared the recognition test accuracies separately for both *implementation status* conditions by means of paired t-tests. Within the previously implemented condition, a significantly higher accuracy on positively instructed than on negatively instructed rules was observed (t(29) = 4.69, *p(t)* < 0.001). Within the not implemented condition there was a mean difference pointing into the same direction but this was only marginally significant (t(29) = 1.85, *p(t)* = 0.075).

#### Correlations between rule implementation and recognition

In order to assess the relationship between both aspects of IBL investigated in our study, we computed the correlation between measures of instruction implementation (RT and switches over the course of the implementation phase) and instruction recognition (recognition test accuracy). As previous analyses (see 2.2.3) emphasized that differences between conditions were mainly driven by alternative-to-alternative switches, correlational analyses specifically focused on that switch type. Since there were no implementation phase RTs in the not implemented condition, implementation phase RTs of actually implemented rules (i.e., the same as in the previously implemented condition) were used separately for each instruction type. All correlation results are summarized in Table 1.

**Table 1:** Experiment 1 – correlation between implementation markers and recognition test accuracy. Asterisks indicate significant pearson correlation coefficients at *p* <.05. Abbreviations: REC = recognition Test; IMP = implementation phase; a2a = alternative-to-alternative; RT = response time; SR = stimulus repetition

		negative				positive			
Variable 1	Variable 2	**SR1**	**SR2**	**SR3**	**SR4**	**SR1**	**SR2**	**SR3**	**SR4**
*REC: implemented*	*IMP: RT*	*r* =.38*	*r* =.46*	*r* =.47*	*r* =.44*	*r* = −.04	*r* = −.08	*r* = −.13	*r* = −.17
	*IMP: a2a-switches*	-	*r* =.18	*r* =.19	*r* =.18	-	*r* = −.70*	*r* = −.69*	*r* = −.71*
*REC: instructed*	*IMP: RT*	*r* =.48*	*r* =.51*	*r* =.48*	*r* =.42*	*r* =.17	*r* =.13	*r* =.04	*r* =.00
	*IMP: a2a-switches*	-	*r* =.29	*r* =.16	*r* =.16	-	*r* = −.49*	*r* = −.54*	*r* = −.57*

**Fig. 5:**
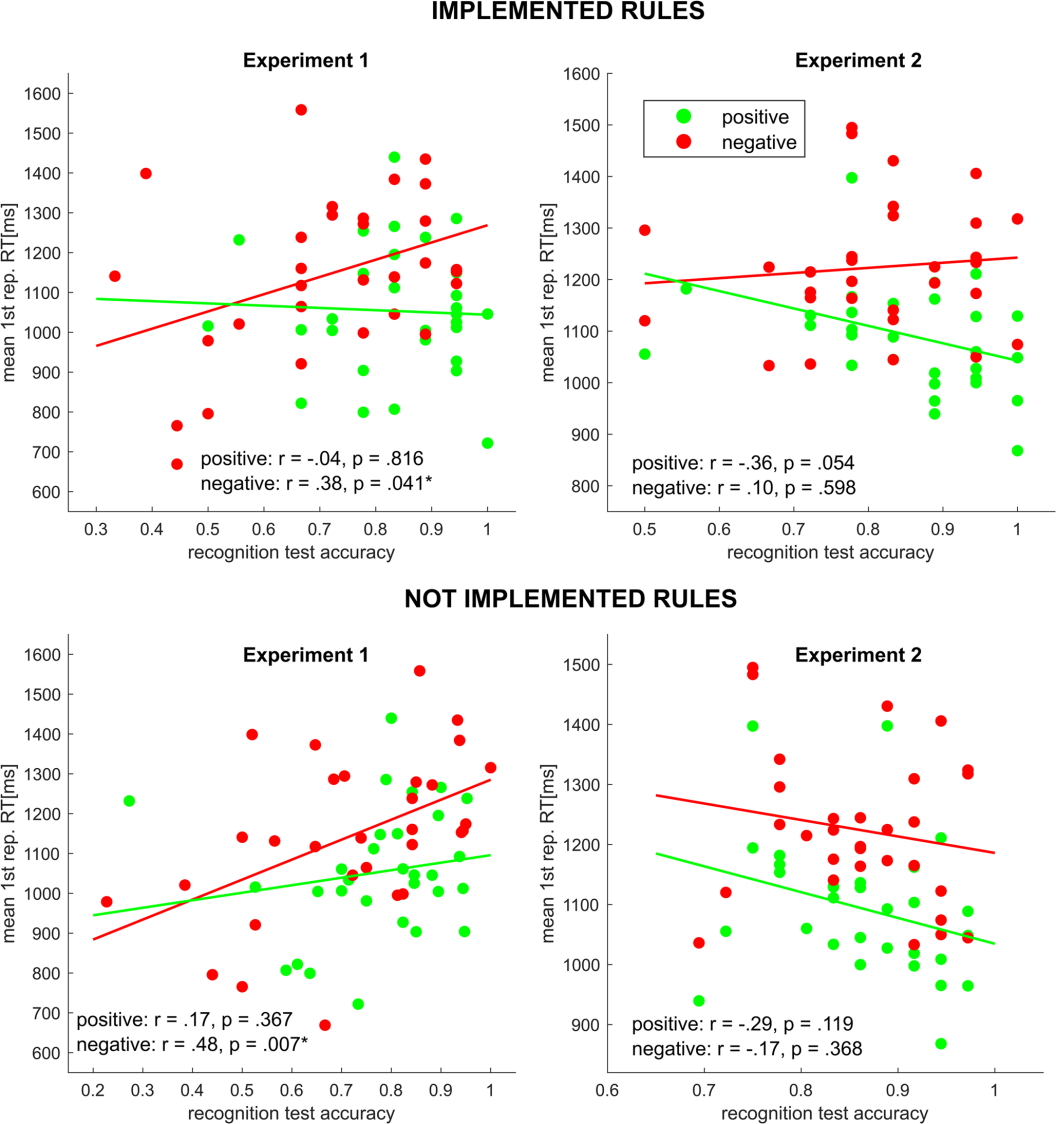
Scatter plots depicting the association between rule implementation (response time at first stimulus repetition) and recognition (accuracy) for implemented and not implemented rules separately for each of the two instruction conditions

We observed a strong positive correlation between implementation RT and recognition test accuracy in the negative condition but not in the positive condition. This pattern was consistent for the recognition test on previously implemented and on not implemented rules. For an exemplary illustration of this pattern, see Fig. 5.

Interestingly, the correlational pattern was quite different when using response switches. Here, we only observed significant negative correlations in the positive condition (i.e., high switching rates were associated with poor recognition). The latter finding was not surprising and rather trivial, given switching tendency in the positive condition is always linked to erroneous implementation behavior most likely rooting in inaccurate encoding which, in turn, should also affect the recognition test.

### Discussion

To summarize the results of Experiment 1, we observed longer RTs in the negative than in the positive instruction condition as well as generally decreasing RTs towards the end of the implementation phase. Yet, the RT difference between both instruction types was constant across stimulus repetitions. Furthermore, we observed a significantly higher switching rate in the negative than in the positive condition. This difference was driven by switches between both alternative (i.e., not instruction-related) response options. In contrast to the RT results, switching rate decreased significantly across repetitions in the negative condition while staying constantly low in the positive condition. This finding was corroborated by the complementary switching analysis (see Supplementary Materials, Sect. 2). The recognition test showed that the instructed S-R associations were generally well remembered across all conditions. Yet, in case of actually implemented rules, the originally instructed S-R rule was less accurately recognized in the negative instruction condition compared to the positive instruction condition. However, for not implemented rules no significant difference was observed between instruction types. Finally, longer implementation RTs were significantly associated with higher recognition accuracy in the negative but not in the positive instruction condition and this was the case for implemented as well as not implemented rules. There was no substantial evidence of a similar association between response switches and recognition accuracy.

For positive instructions previous theorizing posits one key challenge, which is, to transform the instruction from a declarative into a procedural and therefore actionable state (Brass et al., 2017; Ruge et al., 2024). For negative instructions, the transformation into an actionable format additionally and necessarily requires an update of the S-R association’s identity from the instruction-related (negated) S-R association into an affirmative implementation-related S-R association. One of our core questions was whether and to which extent this arguably more complex transformation process would be completed before the first implementation trial.

RT being constantly prolonged from the first to the final implementation trial clearly suggests that transformation of negative instructions into an actionable format is disadvantaged prior to the first implementation trial and continues to be disadvantaged to a similar extent also following repeated implementation trials. This clearly refutes a scenario where the negative instruction is directly encoded as an implementation-related affirmative S-R association equivalent to the implementation-related S-R association following positive instructions. The finding that recognition scores for not implemented S-R rules were comparable between both instruction types further supports the idea that a negated S-R rule was encoded as reliably as an affirmative S-R rule in case of positive instructions. We can thus think of three possible sources for the implementation cost associated with negative relative to positive instructions in Experiment 1: First, prolonged RT in the negative condition might reflect ‘interference cost’ due to a potential conflict between the instruction-related and the implementation-related S-R association (Dudschig & Kaup, 2018). Second, it might additionally reflect actual ‘negation cost’ arising from the time that is consumed by applying the negation operator in order to derive possible implementation-related S-R associations in the first place. Third, additional ‘choice cost’ might arise due to choosing between two alternative response options in the negative condition.

The general RT speed-up across stimulus repetitions suggests increasing fluency at rule execution in both conditions. Interestingly, the RT cost for negative instructions remained constant over the course of the entire implementation phase. Based on previous studies comparing different positive instruction set sizes (Baumann et al., 2023; Ruge et al., 2019) we had hypothesized that initial RT cost induced by increased levels of instructional load (here: negative vs. positive instructions) would become less pronounced towards the end of the implementation phase. Such a pattern was previously interpreted to reflect IBL-induced automatization processes (cf. Mohr et al., 2016) which are slowed down in cognitively more demanding conditions especially during the very first few implementation trials (Baumann et al., 2023). The lack of a practice-related decrease in RT implementation cost associated with negative instructions in turn suggests a more profound and longer lasting impact on rule implementation. The negative condition correlation results seem to support this account to some extent as RT is continuously associated with the key indicator of instruction-related S-R integrity (i.e., recognition accuracy). This points to a different nature of the underlying processes compared to those mediating the impact of higher instructional load such as higher set sizes. While we cannot extrapolate our results beyond the relatively small number of 4 stimulus repetitions, our current finding is reminiscent of long lasting implementation cost in well-practiced negated rules compared to affirmative rules (Dudschig & Kaup, 2018; Wirth et al., 2023). Consistent with their interpretations, our results imply the constant persistence of the instruction-related S-R association across the entire implementation phase. Importantly, unlike those previous studies, in our experiment the negated response option was not explicitly refreshed during actual rule execution as only the stimulus itself was repeated during the implementation phase.

Complementing the analysis of implementation RT, we also examined response switching behavior – as a uniquely meaningful indicator in the negative condition – in order to track implementation dynamics over time. Here, alternative-to-alternative switches were thought to be of particular relevance. Specifically, in the negative instruction condition they reflect purely correct (and goal-directed) behavior whereas in the positive condition they are exclusively related to errors. Thus, we assumed that in the positive condition alternative-to-alternative switches would capture some residual tendency to alternate between eligible response options in the absence of trial-specific feedback rendering them an empirical baseline in this regard. As the overall switching rate difference between instruction conditions was mainly driven by this particular switch type, we can indeed conclude, that switching – between two equally correct response options – reflects a relevant behavioral tendency in the negative condition. As pointed out in the introduction and consistent with the RT results, a parsimonious account for such elevated alternative-to-alternative switching rates would be that rule implementation in the negative condition still relies on the instruction-related S-R association (e.g., B-X). However, alternatively, rule transformation could have taken place in a sense that, based on the negative instruction, two equally strong implementation-related rules would have been formed (i.e., B-Y and B-Z) already during the instruction phase. Switching would then arise from competition between them without the necessity of retrieving the instruction-related S-R association. While such a strong double association seems somewhat unlikely given the constraints of the instruction (i.e., encoding time per S-R association) and the sheer amount of actionable, implementation-related S-R associations that would need to be maintained (i.e., 9 rules per learning block) this account cannot be fully ruled out based on the results of Experiment 1 (but see Experiment 2 which provides evidence against this account).

However, most importantly, and regardless of whether switching was caused by initial retrieval of the instruction-related S-R association or competition between two implementation-related S-R associations switching rates in the negative condition decreased across stimulus repetitions. This clearly indicates that one of the two possible correct implementation-related S-R associations is becoming increasingly distinct and dominant.

This latter observation is somewhat at odds with RT cost remaining constant throughout the implementation phase – as one would assume that an increasingly dominant (i.e., distinct) implementation-related S-R association would also result in a relative RT speed-up. One possible explanation rests on the notion of various potential sources of the implementation cost in the negative condition. As mentioned above, prolonged RT might reflect a mixture of all three types of cost (‘negation’, ‘choice’, and ‘interference’). In turn, alternative-to-alternative response switching might be a more specific indicator of choice cost as it should reflect the competition between the two remaining options *after* the negation operator has already been applied successfully. Based on this assumption, decreasing switching rate differences relative to the empirical positive instruction baseline could be attributed to a reduction in choice cost caused by a more distinct implementation-related S-R association. Consequently, either interference cost or negation cost should become stronger in order to keep total implementation cost – as reflected in RT – constant. There is no reason to assume that negation cost should increase towards the end of the implementation phase – as an applicable implementation-related S-R association should render negation of the instruction-related S-R association less important. Thus, the only remaining and likely contributor is interference cost: At the first few implementation trials, the instruction-related S-R association might be actively retrieved in order to support successful rule implementation as there is no (strong) actionable, implementation-related S-R association (resulting in relatively high negation and choice cost). With the evolution of the latter, rule implementation would become less dependent on such controlled retrieval of the former. However, with the instruction-related representation still present (or the awareness of its importance for the recognition test), there is a growing potential for interference at the S-R level as the implementation phase progresses. In this case, retrieval of the instruction-related S-R association might change its nature and become rather involuntary (and less controlled).

Lastly, an increasingly stable implementation-related S-R association conflicting with the original instruction-related S-R association could also account for significantly lower recognition rates on previously implemented negative instructions. Lower overall recognition test accuracies on not implemented rules of both instruction conditions, in turn, might underlie a general memory decay as they are not reinstated during the implementation phase. Interestingly, the correlation between negative condition recognition test accuracy and implementation RTs could be generalized to not implemented rules as well – numerically even at a greater strength. For previously implemented rules, implementation-specific processes such as reinforcement through repeated retrieval, inhibition or competition with an implementation-related S-R association could drive the relationship between RT and recognition accuracy. In the case of non-implemented rules, an influence of any of these processes can be ruled out. This renders the recognition test accuracy for non-implemented rules the purest indicator of general instruction-related S-R association representation. Thus, the correlation results for non-implemented rules suggests a role of general instruction-related S-R representation during negative rule execution beyond any specific implementation history.

To conclude, Experiment 1 results, especially with respect to the dynamics over the course of the implementation phase, highlight two important aspects: First, in the negative condition the S-R association that is negated during the instruction phase is constantly present as reflected in elevated overall RTs and switching rates across all implementation trials as well as in above chance recognition test accuracy. This is, however, accompanied by the formation of a stable, alternative S-R association at and through rule implementation that becomes increasingly more applicable. Therefore, our results imply the coexistence of two S-R associations involving the same stimulus of which one refers to the instruction and one refers to the implementation of a rule.

However, with respect to instruction-induced rule transformation processes depending on rule type, the interpretability of Experiment 1 might be somewhat limited due to our operationalization of the implementation phase. Specifically, in the implementation phase, color was thought to serve as a retrieval cue for instruction type (i.e., positive or negative) with the stimuli being presented in the same color as during the instruction. This was done in order to keep task difficulty at a reasonable level in general as well as somewhat balanced between instruction conditions. At the same time, strictly speaking, the position of a stimulus (i.e., the associated response key) definitely needed to be encoded during the instruction phase, whereas the color (i.e., the instruction type) might have been encoded only at the first stimulus repetition in the implementation phase. If that was the case, instruction type would not actually have been part of initial instruction encoding. Consequently, this information would not be used in order to proactively prepare implementation or, more generally speaking, not be included in the building of a task model(Duncan et al., 2008) that could be brought into an action-oriented state (cf. Brass et al., 2017). We addressed this issue by conducting Experiment 2 and subsequently comparing both experiments.

## Experiment 2

As mentioned above, Experiment 1 was designed such that participants would have the opportunity to already encode the instruction type (positive or negative) during the instruction phase and before the first implementation trial. With that, they also had the opportunity to proactively engage at least partially in the transformation into actionable implementation-related S-R representations. Our results were clear in showing that this transformation was certainly not complete in the sense that implementing negative instruction still came at a cost relative to positive instructions. However, our result cannot quantify the extent of proactive S-R transformation. It is even possible that no proactive transformation occurred at all. The reason is that instruction type was not only indicated during the instruction phase (green or red color) but also again for each implementation trial. Hence, in principle, participants could entirely ignore the color during instruction and still perform correctly during the implementation phase. In order to quantify the extent to which instruction type was encoded and potentially used for proactive transformation already during the instruction phase in Experiment 1, we conducted Experiment 2, where instruction type was not yet specified during the instruction phase. Hence, any instruction-type specific S-R transformation process could occur only reactively at the time of the first implementation trial. Crucially, if participants had engaged proactively in S-R transformation processes following negative instructions in Exp. 1, this should be reflected by reduced implementation cost relative to Exp. 2.

### Methods

#### Participants

For Experiment 2, 36 new participants (mean age = 22.6, SD age = 2.83; 21 females and 15 males) were recruited. The same inclusion criteria as for Experiment 1 were applied. Participants gave written informed consent in accordance with the Declaration of Helsinki before the start of the experiment and received 10 Euros or were compensated with course credit for their participation. Depending on their performance in the experiment, subjects could gain up to 5 Euro of additional payment (mean = 3.57€; range: 2.25€ to 4.64€).

Based on performance below chance level in the negative condition, 5 subjects were excluded from the analyses. This was also the case for one subject with more than 20% invalid trials. Therefore, the final sample included 30 subjects.

#### Differences between experiments 1 and 2

As Experiment 2 in some sense served as a control experiment with respect to Experiment 1, the general task structure and setup of both experiments was very much alike. The same stimulus material was used and the timing was identical. The key difference, however, was that in the first phase of each learning block in Experiment 2 all 6 stimuli and the vertical bars denoting their relative positions were presented in plain white font instead of colors green and red (indicating positive and negative instructions in Experiment 1, respectively). Two nouns were used per each relative position (left, middle, right) in each learning block. Again, all word-position associations were presented in random order. Importantly, and exactly like in Experiment 1, in the second phase, stimuli were displayed in colored font which indicated whether the key corresponding to the association from the first phase had to be pressed (green font; positive instruction) or whether this exact key was not to be pressed but one of the two alternative ones (red font; negative instruction). Like in Experiment 1, 4 stimuli from the first phase re-appeared in the second phase, each repeated 4 times. Per learning block, 2 stimuli appeared in each of the two colors. The 4 stimuli were selected such that associations with each of the three relative positions occurred at least once and no relative position occurred twice within a color (i.e., instruction type) condition. To prevent subjects from being distracted by the font color of the announcements at the beginning of each experimental phase (e.g., ‘Phase 1: memorize’) these announcements were displayed in yellow in Experiment 2.

#### Analyses

Experiment 2 key analyses largely paralleled the analyses conducted for Experiment 1. As consequence of the stimulus-position associations being displayed in neutral color in the first phase, no distinction between positive and negative instruction types was possible for the subsequent recognition test on not implemented associations. Therefore, recognition test accuracy rates were compared by means of a one-way repeated measures ANOVA with the single within-factor *implementation status* comprising three factor levels (implemented positive, implemented negative, not implemented).

### Results

#### Implementation: accuracy

The 2-by-4 repeated measures ANOVA on implementation phase accuracy rates (see Fig. 2, upper panel) yielded a significant main effect of *instruction type* (F_1,29_ = 36.26; *p(F)* < 0.001; *η*_*p*_^*2*^ = 0.56) indicating higher overall accuracy rates in the negative condition. A marginally significant main effect of *stimulus repetition* (F_2.23,64.68_ = 2.85; *p(F)* = 0.059; *η*_*p*_^*2*^ = 0.09, linear contrast: F_1,29_ = 4.32; *p(F)* = 0.047; *η*_*p*_^*2*^ = 0.13) hinted towards overall increasing accuracy across stimulus repetitions. A more pronounced accuracy rate increase across repetitions in the negative than in the positive condition was indicated by a significant interaction of *instruction type* x *stimulus repetition* (F_3,87_ = 6.60; *p(F)* < 0.001; *η*_*p*_^*2*^ = 0.19, linear contrast: F_1,29_ = 10.15; *p(F)* = 0.003; *η*_*p*_^*2*^ = 0.26). As in Experiment 1, the instruction type difference most likely reflects the chance level imbalance between the positive and the negative condition and should thus be interpreted cautiously.

#### Implementation: response time

Significant main effects of *instruction type* (F_1,29_ = 158.80; *p(F)* < 0.001; *η*_*p*_^*2*^ = 0.85) and *stimulus repetition* (F_1.83,52.93_ = 151.35; *p(F)* < 0.001; *η*_*p*_^*2*^ = 0.84, linear contrast: F_1,29_ = 249.13; *p(F)* < 0.001; *η*_*p*_^*2*^ = 0.90) were observed indicating longer overall RTs in the negative condition and decreasing overall RTs across repetitions, respectively. Replicating Experiment 1, no significant interaction of *instruction type* x *stimulus repetition* was observed (F_3,87_ = 0.74; *p(F)* = 0.974; *η*_*p*_^*2*^ < 0.01, linear contrast: F_1,29_ < 0.01; *p(F)* = 0.951; *η*_*p*_^*2*^ < 0.01). For an illustration, see Fig. 2 (lower panel).

#### Implementation: response switches

The 2-by-3-by-3 repeated measures ANOVA on response switches (see Fig. 3) yielded a significant main effect of *instruction type* (F_1,29_ = 229.34; *p(F)* < 0.001; *η*_*p*_^*2*^ = 0.88) indicating a greater overall tendency to alternate between response options in the negative condition. Furthermore, the overall switch tendency was decreasing towards the end of the implementation phase (F_2,58_ = 21.23; *p(F)* < 0.001; *η*_*p*_^*2*^ = 0.42, linear contrast: F_1,29_ =31.96; *p(F)* < 0.001; *η*_*p*_^*2*^ = 0.52) as indicated by a significant main effect of *stimulus repetition*.

Also, again replicating Experiment 1, we found a more pronounced switch probability decrease across repetitions in the negative as compared to the positive condition which was indicated by the significant interaction of *instruction type* x *stimulus repetition* (F_2,58_ = 3.74; *p(F)* = 0.030; *η*_*p*_^*2*^ = 0.11, linear contrast: F_1,29_ = 5.33; *p(F)* = 0.028; *η*_*p*_^*2*^ = 0.16). However, follow-up ANOVAs performed analogously to Experiment 1, yielded a significant main effect of *stimulus repetition* – indicating decreasing switching rates – for both, the negative as well as the positive instruction type (F_2,58_ = 14.79; *p(F)* < 0.001; *η*_*p*_^*2*^ = 0.34, linear contrast: F_1,29_ =22.35; *p(F)* < 0.001; *η*_*p*_^*2*^ = 0.44 and F_1.64,47.43_ = 8.29; *p(F)* = 0.002; *η*_*p*_^*2*^ = 0.22, linear contrast: F_1,29_ =11.23; *p(F)* = 0.002; *η*_*p*_^*2*^ = 0.28, respectively).

Furthermore, a significant main effect of *switch type* (F_1.19,34.55_ = 12.54; *p(F)* < 0.001; *η*_*p*_^*2*^ = 0.30) was observed, indicating a higher overall rate of alternative-to-alternative switches compared to both instructed-to-alternative (t(29) = 3.20, *p(t)* = 0.010) and alternative-to-instructed (t(29) = 3.95, *p(t)* = 0.001), while the latter two types did not differ significantly from each other (t(29) = 2.34, *p(t)* = 0.080). Like in Experiment 1, there was also a significant interaction of *instruction type x switch type* (F_1.27,36.84_ = 83.69; *p(F)* < 0.001; *η*_*p*_^*2*^ = 0.74). Unlike in Experiment 1, paired t-tests revealed significantly higher switching rates for the negative than for the positive instruction type at all switch types (all t(29) > 3.62, all *p(t)* < 0.002). In the negative condition, alternative-to-alternative switches were significantly more frequent then instructed-to-alternative switches (t(29) = 6.41, *p(t)* < 0.001), and both were more frequent than alternative-to-instructed switches (t(29) = 7.15, *p(t)* < 0.001 and t(29) = 4.34, *p(t)* < 0.001, respectively). Like in Experiment 1, in the positive condition, alternative-to-alternative switches were significantly less frequent then both instructed-to-alternative (t(29) = −6.96, *p(t)* < 0.001) and alternative-to-instructed switches (t(29) = −9.36, *p(t)* < 0.001) whereas the latter types did not differ from each other (t(29) = 0.60, *p(t)* > 0.999).

As in Experiment 1, neither a significant interaction *switch type x stimulus repetition* (F_4,116_ = 1.51; *p(F)* = 0.204; *η*_*p*_^*2*^ = 0.05, linear contrast: F_1,29_ = 1.23; *p(F)* = 0.276; *η*_*p*_^*2*^ = 0.04) nor a significant three-way interaction *instruction type x switch type x stimulus repetition* (F_4,116_ = 0.88; *p(F)* = 0.476; *η*_*p*_^*2*^ = 0.03, linear contrast: F_1,29_ < 0.01; *p(F)* = 0.98; *η*_*p*_^*2*^ < 0.01) were detected.

##### Alternative-to-alternative switches

Consistent with Experiment we conducted a follow-up ANOVA focusing exclusively on alternative-to-alternative switches. As expected, switching rate was generally higher in the negative than in the positive condition and generally decreased across stimulus repetitions as indicated by main effects of *instruction type* (F_1,29_ = 175.55; *p(F)* < 0.001; *η*_*p*_^*2*^ = 0.86) and *stimulus repetition* (F_1.59,46.16_ = 5.69; *p(F)* = 0.010; *η*_*p*_^*2*^ = 0.16, linear contrast: F_1,29_ = 7.67; *p(F)* = 0.010; *η*_*p*_^*2*^ = 0.21), respectively. The interaction effect *type x repetition* did not reach statistical significance (F_1.60,46.58_ = 1.94; *p(F)* = 0.153; *η*_*p*_^*2*^ = 0.06, linear contrast: F_1,29_ = 2.25; *p(F)* = 0.144; *η*_*p*_^*2*^ = 0.07). As in Experiment 1, switch proportion in consecutively correct repetition instances decreased across the implementation phase in the negative condition (see Supplementary Materials, Sect. 2).

#### Recognition test: accuracy

In each condition (i.e., implemented positive, implemented negative and not implemented), subjects performed significantly above chance level (all t(29) < 13.43, all p(t) < 0.001). Comparing the 3 conditions, the main effect of *implementation status* only reached marginal significance (F_1,29_ =2.94; *p(F)* < 0.061; *η*_*p*_^*2*^ = 0.09). Despite the non-significant effect of *implementation status*, we performed follow-up paired t-tests as – based on Experiment 1 results and relative to the implemented positive condition – we had expected a worse recognition performance in both the implemented negative and the not implemented condition. However, these tests only revealed a significantly lower recognition test accuracy for implemented negative than for not implemented rules (t(29) = −2.56, *p(t)* = 0.047). Implemented positive rules neither significantly differed from implemented negative (t(29) = 1.47, *p(t)* = 0.456) nor from not implemented rules (t(29) = −0.71, *p(t)* < 0.999). For an illustration, see Fig. 4.

#### Correlations between rule implementation and recognition

As for Experiment 1, we examined the relationship between rule implementation (RTs and alternative-to-alternative switches) and rule recognition (accuracy). Note, that for the recognition test on not implemented rules no differentiation between (instruction) types was possible. For an overview, see Table 2.

**Table 2:** Experiment 2 – correlation between implementation markers and recognition test accuracy. Asterisks indicate significant pearson correlation coefficients at *p* <.05. Abbreviations: REC = recognition Test; IMP = implementation phase; a2a = alternative-to-alternative; RT = response time; SR = stimulus repetition

		negative				positive			
Variable 1	Variable 2	**SR1**	**SR2**	**SR3**	**SR4**	**SR1**	**SR2**	**SR3**	**SR4**
*REC: implemented*	*IMP: RT*	*r* =.10	*r* =.16	*r* =.14	*r* =.22	*r* = −.36	*r* = −.11	*r* = −.24	*r* = −.04
	*IMP: a2a-switches*	-	*r* =.00	*r* = −.09	*r* =.12	-	*r* = −.53*	*r* = −.60*	*r* = −.46*
*REC: instructed*	*IMP: RT*	*r* = −.17	*r* =.04	*r* =.01	*r* =.09	*r* = −.29	*r* =.06	*r* = −.04	*r* =.12
	*IMP: a2a-switches*	-	*r* =.16	*r* =.24	*r* =.18	-	*r* = −.54*	*r* = −.49*	*r* = −.26

The only pattern observed consistently (across stimulus repetitions) was a negative relationship between response switches and recognition test accuracy in the positive condition. Again, as in Experiment 1, this emphasizes that positive condition switching is linked to erroneous behavior – due to unsuccessful encoding or maintenance of the instructed S-R. Remarkably, and different from the correlation results of Experiment 1, no significant association between RT and recognition test accuracy was observed in the negative condition at any stimulus repetition (for an exemplary illustration, see Fig. 5).

## Comparison between experiments 1 and 2

To summarize, the results of Experiment 2 were qualitatively similar to the results found Experiment 1 in many aspects. This resemblance was particularly evident in the implementation phase results. In contrast, many correlational findings were not replicated between experiments. An explicit quantitative comparison of both experiments can be found in this section.

Implementation phase accuracy rates, response times and response switches were compared between Experiments 1 and 2 by means of mixed ANOVAs. Besides the already familiar within-subjects factors *instruction type*,* switch type* (for switch-related analyses) and *stimulus repetitions*, the between-subjects factor *experiment* (Experiment 1 versus Experiment 2) was included. Regarding the comparison of recognition test accuracy rates, we again included the between-subjects factor *experiment* and the single within-subjects factor *implementation status* comprising three levels (implemented positive, implemented negative and not implemented). Note, that Experiment 1 recognition test accuracy rates for the not implemented condition were aggregated across positive and negative instructions in order to enable comparability between experiments.

### Results

#### Implementation: accuracy

Neither the main effect of the between-subjects factor *experiment* (F_1,58_ = 0.56; *p(F)* = 0.458; *η*_*p*_^*2*^ = 0.01) nor any of the interactions involving the factor *experiment* reached statistical significance (all F < 1.34; all *p(F)* > 0.251; all *η*_*p*_^*2*^ = 0.03, linear contrast: all F < 2.79; all *p(F)* > 0.100; *η*_*p*_^*2*^ < 0.05).

#### Implementation: response times

A significant main effect of *experiment* (F_1,58_ = 4.77; *p(F)* = 0.033; *η*_*p*_^*2*^ = 0.08) indicated higher overall RTs in Experiment 2 than in Experiment 1. Furthermore, a significant interaction of *instruction type* x *experiment* (F_1,58_ = 4.71; *p(F)* = 0.034; *η*_*p*_^*2*^ = 0.08) indicated a greater RT difference between conditions in Experiment 2 as compared to experiment 1. Neither the interaction *stimulus repetition* x *experiment* (F_1.65,95.78_ = 2.22; *p(F)* = 0.123; *η*_*p*_^*2*^ = 0.04, linear contrast: F_1,58_ = 1.68; *p(F)* = 0.200; *η*_*p*_^*2*^ = 0.03). nor the three-way interaction *instruction type* x *stimulus repetition* x *experiment* (F_3,174_ = 0.08; *p(F)* = 0.970; *η*_*p*_^*2*^ < 0.01, linear contrast: F_1,58_ < 0.01; *p(F)* = 0.976; *η*_*p*_^*2*^ < 0.01) reached significance.

As there was a significant interaction effect of *experiment x instruction* type, we conducted follow-up analyses in order to qualify this interaction effect. Per instruction type condition (i.e., positive and negative) a one-way ANOVA was performed, including only the between-subjects factor *experiment*. In the positive condition, this effect was only marginally significant (F_1,58_ = 3.54; *p(F)* = 0.065; *η*_*p*_^*2*^ = 0.06) whereas in the negative condition, significantly higher overall RTs in Experiment 2 as compared to Experiment 1 were indicated (F_1,58_ = 5.56; *p(F)* = 0.022; *η*_*p*_^*2*^ = 0.09).

#### Implementation: response switches

Two significant effects involving the between-subjects factor experiment were observed. The interaction of *instruction type x experiment* (F_1,58_ = 8.80; *p(F)* = 0.004; *η*_*p*_^*2*^ = 0.13) indicated a more pronounced difference between instruction types in Experiment 2 than in Experiment 1. However, t-tests showed that, between experiments, overall switching rates neither differed from each other significantly in the negative (t(58) = 1.66, *p(t)* = 0.101) nor in the positive (t(58) = −0.75, *p(t)* = 0.459) instruction condition.

Furthermore, the significant interaction of *switch type x experiment* (F_1.23,71.52_ = 4.43; *p(F)* = 0.031; *η*_*p*_^*2*^ = 0.07) seemed to be driven mostly by a higher proportion of alternative-to-alternative switches in Experiment 2 compared to Experiment 1. The according t-test was marginally significant (t(58) = 1.86, *p(t)* = 0.069), whereas no such tendency could be observed for instructed-to-alternative (t(58) = 0.14, *p(t)* = 0.885) and alternative-to-instructed (t(58) = −0.27, *p(t)* = 0.786).

Neither the main effect nor any of the remaining interactions involving *experiment* reached significance (all F < 1.75; *p(F)* > 0.188; *η*_*p*_^*2*^ < 0.04).

##### Alternative-to-alternative switches

As for the separate analyses on Experiments 1 and 2, we conducted a follow-up analysis focusing exclusively on alternative-to-alternative switches. A marginally significant main effect of the between-subjects factor *experiment* (F_1,58_ = 3.44; *p(F)* = 0.069; *η*_*p*_^*2*^ = 0.06) was observed. Importantly, there was a significant interaction of *instruction type x experiment* (F_1,58_ = 6.42; *p(F)* = 0.014; *η*_*p*_^*2*^ = 0.10). This interaction effect could be qualified by t-tests: In the negative condition, switching rate was significantly higher in Experiment 2 than in Experiment 1 (t(58) = 2.37, *p(t)* = 0.021). In the positive condition, there was no significant difference between experiments (t(58) = −0.25, *p(t)* = 0.803). No significant interaction effect of *stimulus repetition x experiment* (F_2,116_ = 0.24; *p(F)* = 0.784; *η*_*p*_^*2*^ < 0.01) was observed. At last, across both experiments, the significant interaction effect *instruction type x stimulus repetition* (F_2,116_ = 3.93; *p(F)* = 0.022; *η*_*p*_^*2*^ = 0.06; linear contrast: F_1,58_ = 6.27; *p(F)* = 0.015; *η*_*p*_^*2*^ = 0.10) indicated a more pronounced switching decrease in the negative than in the positive condition. Importantly, this effect was not further modulated by the between-subjects factor as suggested by a non-significant interaction *instruction type x stimulus repetition x experiment* (F_2,116_ = 0.31; *p(F)* = 0.736; *η*_*p*_^*2*^ < 0.01; linear contrast: F_1,58_ = 0.03; *p(F)* = 0.858; *η*_*p*_^*2*^ < 0.01).

#### Recognition test: accuracy

The 2-by-3 repeated measures ANOVA on recognition test accuracy rates revealed a significant main effect of the between-subjects factor *experiment* (F_1,58_ = 6.08; *p(F)* = 0.017; *η*_*p*_^*2*^ = 0.10). This indicated higher overall accuracy in Experiment 2 than in Experiment 1. In addition, a significant interaction of *experiment* x *implementation status* was observed (F_1,58_ = 6.47; *p(F)* = 0.002; *η*_*p*_^*2*^ = 0.10). This interaction can be further characterized best by two-sample t-tests: Recognition accuracies were significantly higher in Experiment 2 as compared to Experiment 1 in the implemented negative condition (t(58) = 2.39, *p(t)* = 0.020) and in the not implemented condition (t(58) = 3.64, *p(t)* < 0.001). However, this was not the case in the implemented positive condition (t(58) = 0.23, *p(t)* = 0.819). Finally, separately conducted two-sample t-tests corroborated greater Experiment 2 recognition test accuracy on not implemented rules compared to both not implemented rule types from Experiment 1 (positive: t(58) = 2.90, *p(t)* = 0.005; negative: t(58) = 3.63, *p(t)* < 0.001).

### Discussion

The results patterns of Experiments 1 and 2 are structurally very similar but differences between instruction conditions were more pronounced in Experiment 2 than in Experiment 1. While implementation RTs were generally higher in Experiment 2 than in Experiment 1, this difference was significantly more pronounced in the negative condition. A similar result was found for response switches: Negative (but not positive) alternative-to-alternative switches were more frequent in Experiment 2 than in Experiment 1. Finally, in contrast to implementation costs observed for Experiment 2 relative to Experiment 1, recognition test accuracies were significantly higher in Experiment 2 than in Experiment 1, both for negative and for not implemented rules.

The core question that was addressed by the comparison between Experiments 1 and 2 was whether negative instructions presented in Experiment 1 would induce implementation-related transformation processes already during the instruction phase comparable to those observed for regular positive instructions (e.g., Brass et al., 2017; Ruge & Wolfensteller, 2010). If yes, this should be reflected by an implementation benefit in Experiment 1 relative to Experiment 2. Experiment 2 was explicitly designed such that it only differed from Experiment 1 in the way the rules were presented during the initial instruction phase of a new learning block. Thus, behavioral differences between experiments can mainly be attributed to whether instruction-related S-R mappings did (Experiment 1) or did not (Experiment 2) allow for deriving implementation-related response options for future use. Furthermore, the experiment comparison also speaks to the interpretability of response switching behavior. Precisely, in Experiment 1, elevated negative switching rates could either be driven by initial retrieval of the instruction-related S-R association (e.g., A-X) from which both response options would be generated with similar probability or by the competition of two equally strong implementation-related S-R associations involving the alternative response options (i.e., A-Y and A-Z). In the negative condition of Experiment 2, the generation of any such implementation-related S-R association before the first actual implementation trial can reasonably be ruled out. Thus, correct rule application – especially in early implementation phase trials – necessarily depends on retrieving the instruction-related S-R association. The results indicate that under such circumstances (i.e., Experiment 2) negative condition switches occur at an even higher frequency. Therefore, it seems reasonable to conclude that, in Experiment 1 as well, overall switching magnitude reflects the degree to which behavior relies on the instruction-related S-R association (or, conversely, on *one* distinct implementation-related S-R association). As the experiment comparison shows, this happens to a lesser degree in Experiment 1 than Experiment 2.

The comparison results clearly showed that behavioral efficiency as measured by RT significantly benefitted from advance knowledge of instruction type during the instruction phase of Experiment 1 especially in the negative condition and to a significantly lesser extent in the positive condition.

A first explanatory account would be that the implementation benefit of negative instructions in Experiment 1 relative to Experiment 2 is really a direct benefit of making active use of the negation operator already before first-time implementation. In other words, the negative instruction (i.e., the combination of S-R association and negation operator) is assumed to induce an advance transformation process instead of a full two-staged process of first merely encoding the instruction-related S-R association and secondly applying the negation operator later on at the moment of future implementation. Hence, similar to positive instructions in Experiment 1 an action-oriented representation of one or both of the to-be implemented S-R associations might be derived at least partially already around the time of instruction. Switching rate analyses seem to support this idea as they indicate a greater relative reliance on one distinct implementation-related S-R association in Experiment 1. According to this account, positive and negative instructions undergo a qualitatively similar transformation process. Despite this processual similarity, the implementation benefit of positive instructions relative to negative instructions in Experiment 1 could be explained by the difference in representational strength (or completeness of proceduralization) due to the more direct nature of positive instructions (Pereg & Meiran, 2021) and by the absence of time-consuming choice between two correct response options in the negative condition.

A second account would be that the implementation benefit induced by negative instructions is of a more indirect nature. Specifically, neutral instructions from Experiment 2 might be unintentionally encoded in a similar manner as a positive instruction is encoded in Experiment 1. This includes the tendency to start S-R rule proceduralization before first-time implementation. If such an already (partially) proceduralized neutral rule becomes negated at implementation, it needs to be inhibited which in turn prolongs RT. It might seem unfeasible to intentionally produce such an action-oriented rule state given the considerable probability (50%) of harming future implementation in case of negation. However, the role of implementation intention as a prerequisite for IBL has been called into question(Liefooghe et al., 2012; but also Liefooghe & Houwer, 2018) rendering ‘automatic’ action-oriented encoding of the neutral rule a possible option. To conclude, according to this account, the apparent implementation *benefit* of negative instructions in experiment 1 compared to experiment 2 would at least in part rather reflect a relative implementation *cost* in experiment 2 due to the need to overcome this bias in advance proceduralization.

In summary, two major messages could be conveyed by negative instructions: On the one hand, negative instructions might introduce the need and possibility to immediately generate (at least one) future implementation-related S-R association. On the other hand, they might reasonably rule out any implementation probability for the instruction-related S-R association, stopping its harmful proceduralization. Interestingly, at least in the present study, the relative impact of the instruction type information was greater in the negative than in positive condition. While the size of the respective effect was rather small this is still somewhat remarkable. Given the theoretical considerations and empirical findings on the efficiency of proactively prepared direct (and thus) positive instructions (Cole et al., 2017, 2018; Pereg & Meiran, 2021), one could have expected the positive instruction condition from Experiment 1 to most clearly benefit, as it entails an unambiguous S-R association that can be prepared for execution immediately upon encounter.

While the experiment comparison revealed an implementation benefit of Experiment 1 over Experiment 2 in terms of efficiency (at comparable implementation accuracy rates) it also clearly showed a disadvantage with respect to recognition test accuracies. This is particularly interesting, as we expected the recognition test to capture a shared element between both experiments, namely the *instruction-related* S-R association void of any instruction type information.

Assuming that the instruction-related S-R association is bearing a greater relevance during negative rule implementation in Experiment 2 than in Experiment 1 the repeated retrieval of this S-R association in order to generate a response for implementation would most likely strengthen its representation and thereby imply better recognition test performance.

The strong recognition performance advantage for experiment 2 with respect to not implemented rules calls for a more detailed discussion. Importantly, this advantage was observed when contrasting not implemented neutral S-R rules from Experiment 2 with both not implemented positive instructions and not implemented negative instructions from Experiment (1) There are two explanatory accounts of this observation that seem plausible to us: First, and very basically, the lack of the additional instruction type component during the instruction phase in Experiment 2 might have resulted in a less complex rule which could have been more easily encoded, memorized and maintained. It is, however, difficult to see how such facilitated memorization should not also have resulted in a significantly higher accuracy at the level of actual rule implementation (which was not the case). Second, as mentioned above, the neutral stimulus-position association presented in Experiment 2 does not reasonably invite for any kind of actual response preparation (even though there might be a tendency to unintentionally prepare a positively instructed response). Therefore, the neutral rules that remained unimplemented until the recognition test might have been stored in a format that is optimized for later recognition - perhaps resembling stimulus-stimulus (S-S) associations(cf. Tibboel et al., 2016) rather than an actual S-R associations. This is consistent with previous findings in the IBL domain that have emphasized an advantage of merely instructed associations over instructed and concurrently implemented rules in some (explicit) test contexts (Jargow et al., 2022; Pfeuffer et al., 2017; Ruge et al., 2024). Such kind of differences in the way instruction-related S-R associations were stored might – alongside a general reduction in variance in Experiment 2 – be the reason why several correlational patterns from Experiment 1 could not be replicated in Experiment (2) In general, correlation results from both, Experiment 1 and Experiment 2 served exploratory purposes and should thus be interpreted cautiously, especially given the sample size of *N* = 30 (cf. Schönbrodt & Perugini, 2013).

To conclude, the experiment comparison showed that although the experimental setup in Experiment 1 allowed for separate and sequential encoding of the instruction components (i.e., stimulus-position association during the instruction phase and instruction type during the implementation phase) this feature was not exploited. Rather, implementation efficiency benefitted from an instruction that entails specification with respect to an S-R association’s future use. The nature of this benefit in the negative condition might rest on proactive preparation of the implementation-related S-R association or the absence of unintentional instruction-related S-R association proceduralization. Further, the recognition test results imply that there are differences in the way the instruction-related S-R association is stored depending on whether or not it can directly be used in order to derive a response (regardless of the instruction type).

## Experiment 3

The comparison between Experiments 1 and 2 revealed that knowing instruction type in advance had a significant impact on both implementation and recognition of novel S-R rules compared to ad-hoc affirmation or negation, respectively. Although Experiment 3 was primarily designed for functional magnetic resonance imaging (fMRI) purposes, we decided to include a report of the behavioral results in the present paper in order to further substantiate the results obtained in Experiment 1 based on a larger sample and by addressing some aspects in experimental design that could have impacted Experiment 1 results.

### Methods

#### Sample

Fifty-five subjects (mean age: 22.98, 36 females and 19 males) were recruited from the subject pool of Technische Universität Dresden. Due to incomplete data collection, 3 subjects were excluded. Consistent with Experiment 1 and 2, another two subjects were excluded due to below chance-level accuracy in phase 2 of the negative instruction condition. Thus, the final sample size for all behavioral analyses was at *N* = 50.

All participants were right-handed, neurologically healthy, and had normal or corrected-to-normal vision. All subjects reported to not suffer from any kind of color blindness prior to participation. However, two subjects notified the experimenter about problems with perception of the blue color tone used in one of the experimental conditions after the training blocks. Therefore, for those two subjects an experimental version was used in which the blue color tone was replaced by a green one.

The experimental protocol was previously approved by the Ethics Committee of the Technische Universität Dresden (EK586122019) and conformed to the World Medical Association’s Declaration of Helsinki. All participants gave written consent before taking part in the experiment and were paid 12 Euro per hour or were compensated with course credit for their participation. Depending on their performance in the experimental task, subjects could gain up to 5 Euro of additional payment (mean = 3.82€; range: 2.12€ to 4.75€).

#### Changes with respect to experiment 1

The experiment took place inside an MRI-scanner. Stimuli were projected onto a screen behind the scanner which was viewed by participants through a rear-facing mirror. An MRI-compatible keyboard with four keys was used for responding. Subjects were asked to place the index, middle, and ring finger of their right hand onto first, second and third key from the left, respectively.

Several changes to the paradigm were necessary due to the fMRI setup. First there were no announcements at the start of each individual phase (instruction, implementation recognition test) which effectively resulted in a direct transition between experimental phases. Instead of the announcements, instructions screens were marked by black rectangular frames whereas implementation screens were marked by white rectangular frames. No frame was displayed for the recognition test. Furthermore, all instructions in phase 1 remained on screen for 1500ms (instead of 2000ms in Experiment 1) and the response window for the recognition test was cut to 5000ms (instead of 10000ms in Experiment 1). Also, there was no feedback after the implementation phase anymore but only the general feedback at the end of each learning block (see below). All of these changes were implemented to keep the time inside the scanner as short as possible. Second, an inter-trial interval (ITI) was inserted before and after each instruction trial, each implementation trial, and each recognition test trial. During the ITI a white fixation cross was presented centrally on screen. The ITI duration varied randomly within a range from 2 to 4 s (in steps of 500 ms). This was necessary in order to enable estimation of trial-related brain activation versus baseline. Third, the type of multivariate pattern analysis that was planned for the functional imaging data(cf. Fregni et al., 2024) required a change of the stimulus sequence in the implementation phase: In Experiment 3, stimuli were presented in a pseudo-random order such that each stimulus was presented for the i^th^ time before another stimulus was presented for the i + 1th time (e.g., all to-be implemented stimuli were presented for the first time before any stimulus was presented for the second time). Different from Experiments 1 and 2, this setup allowed for direct repetitions of the same stimulus.

Besides these fMRI-related adaptations, we made two additional, deliberate changes: First, instead of the colors green and red we now used the colors blue and orange to indicate instruction type. The assignment of color to instruction type was counterbalanced across subjects and remained constant across the entire experiment. This was done, in order to rule out a potential confounding of instruction condition with instruction color. Second, the style in which the S-R mappings (re-)appeared in the recognition test was changed: Stimuli were now accompanied by one of three positional attributes (the German word for ‘left’, ’middle’, or ‘right’) below them but not by vertical bars. These attributes either did or did not match the relative position of the stimulus word between the two vertical bars during the instruction. This change was implemented to minimize the possibility of mere visual matching between the instruction and recognition test phase. Furthermore, the left (index finger) and right (ring finger) key had to be used for ‘No’ (match) and ‘Yes’ (non-match) responses, respectively.

Each learning block began with an on-screen prompt indicating the current block number (‘Start: Block X/36’) displayed for 1500 ms. It concluded with a feedback screen showing participants’ accuracy in phase two (‘Execute: X/16’ at the top), phase three (‘Recognition Test: X/2’ in the middle), and their total points for the block (‘Points: X’ at the bottom) that was shown for 2500 ms.

Before the start of the actual experiment, the experimental task was practiced in 4 learning blocks outside the scanner. After receiving general task instructions, subjects completed two learning blocks that slightly deviated from the main experiment: Before the start each of the three phases, there was an announcement specifying the meaning of the current phase, with the phase number and the German words for ‘memorize’, ‘execute’ and ‘recognition test’ being displayed centrally on screen for 1500ms. After two blocks, subjects were informed that the announcements would neither be part of the remaining training blocks nor of the main experiment inside the scanner.

#### Power considerations and statistical analysis

No power analyses were conducted for Experiment 1. The reason was that due to the novelty of the experimental approach the expected effect sizes could not be quantified objectively. Experiment 3 constitutes a conceptual replication of Experiment 1. Thus, effect sizes of the latter were used to conduct power calculations for the former using GPower (Version 3.1.9.7; Faul et al., 2009).

For a very large effect like the basic RT difference between instruction conditions (Cohen’s f = 1.42 in Experiment 1) a power of 0.99 would be achieved with a sample size of *N* = 5. For a relatively modest effect like the interaction of instruction type by implementation status on recognition test accuracy (Cohen’s f = 0.40 in Experiment 1) a power of 0.99 would be achieved with a sample size of *N* = 31. At last, at the lower end of observed effect sizes, for a correlation of *r* = .38 (between RT and recognition test for unimplemented stimuli in Experiment 1, one-tailed) a power of 0.80 would be achieved with a sample size of *N* = 41. Hence, with a sample size of *N* = 50, Experiment 3 is well-powered to identify the most relevant effects.

Experiment 3 analyses paralleled Experiment 1 analyses with one exception. For the analysis of response switches we only included the alternative-to-alternative category. As elaborated on above, this switching category is the most relevant to our hypotheses and it can be interpreted in a straight-forward way as it is not directly influenced by an overall increase in implementation accuracy across stimulus repetitions. Analyses were carried out with IBM SPSS 28, Matlab 2018b and R.

### Results

#### Implementation: accuracy

**Fig. 6:**
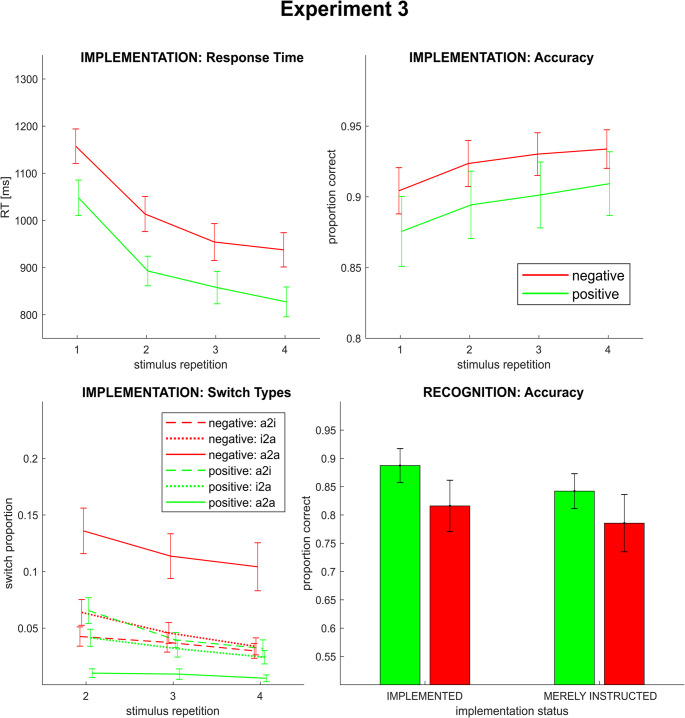
Experiment 3 implementation phase RT (upper left panel), accuracy (upper right panel), switches (lower left panel) and recognition test accuracy (lower right panel). Error bars represent 95% confidence intervals

As for Experiments 1 and 2, a significant main effect of *instruction type* indicated higher overall implementation accuracy on negatively compared to positively instructed rules (F_1,49_ = 13.21; *p(F)* < 0.001; *η*_*p*_^*2*^ = 0.21). Again, this should be interpreted cautiously, as it most likely reflects a chance-level imbalance due to different number of response options. Again replicating Exp. 1, no significant interaction of *instruction type x stimulus repetition* (F_2.66,130.36_ = 0.23; *p(F)* = 0.875; η² = 0.01, linear contrast: F_1,49_ = 0.31; *p(F)* = 0.580; *η*_*p*_^*2*^ = 0.01) was observed. Different from Experiment 1, we also observed a significant main effect of *stimulus repetition* indicating increasing accuracy rates over the course of the implementation phase (F_2.40,117.80_ = 20.48; *p(F)* < 0.001; *η*_*p*_^*2*^ = 0.29, linear contrast: F_1,49_ = 43.11; *p(F)* < 0.001; *η*_*p*_^*2*^ = 0.47). For an illustration, see Fig. 6, upper right panel.

#### Implementation: response time

In line with Experiments 1 and 2, we observed a significant main effect of *instruction type* indicating higher overall RTs during the implementation of negatively instructed rules as compared to positive ones (F_1,49_ = 183.32; *p(F)* < 0.001; *η*_*p*_^*2*^ = 0.79). Furthermore, there again was an overall RT decrease as indicated by a significant main effect of *stimulus repetition* (F_1.70,83.09_ = 354.35; *p(F)* < 0.001; *η*_*p*_^*2*^ = 0.88, linear contrast: F_1,49_ = 461.01; *p(F)* < 0.001; *η*_*p*_^*2*^ = 0.90). A marginally significant interaction effect of *instruction type x stimulus* repetition was detected *(*F_3,147_ *= 2.48; p(F) = 0.070; η*_*p*_^*2*^ *= 0.05*). Like in Experiments 1 and 2, however, this interaction did not follow a linear trend (linear contrast: F_1,49_ = 0.73; *p(F)* = 0.396; *η*_*p*_^*2*^ = 0.02). For an illustration, see Fig. 6, upper left panel.

#### Implementation: response switches

Paralleling Experiments 1 and 2, alternative-to-alternative response switches (Fig. 6, lower left panel) were more frequent in the negative than in the positive instruction condition (F_1,49_ = 117.37; *p(F)* < 0.001; *η*_*p*_^*2*^ = 0.71) as well as generally less frequent towards the end of the implementation phase (F_2,98_ = 15.01; *p(F)* < 0.001; *η*_*p*_^*2*^ = 0.23, linear contrast: F_1,49_ = 24.99; *p(F)* < 0.001; *η*_*p*_^*2*^ = 0.34).

In line with both previous experiments and our hypotheses, we observed a significant interaction effect of *instruction type x stimulus repetition* indicating a more pronounced switching rate decrease across repetitions in the negative than in the positive condition (F_1.77,86.71_ = 8.38; *p(F)* < 0.001; *η*_*p*_^*2*^ = 0.15, linear contrast F_1,49_ = 12.94; *p(F)* < 0.001; *η*_*p*_^*2*^ = 0.21). As in Experiment 1, this is consistent with a linearly decreasing switch proportion in consecutively correct repetition instances across the implementation phase in the negative condition (see Supplementary Materials, Sect. 2).

Please note, that although statistical analysis in Experiment 3 focused on alternative-to-alternative switches, all switch types are shown in Fig. 6 (lower left panel) for completeness.

#### Recognition test: accuracy

As in Experiments 1 and 2, recognition test accuracy (Fig. 6, lower right panel) was well above chance-level in all experimental conditions (all t(49) > 11.06; all *p(t)* < 0.001).

Consistent with Experiment 1, a significant main effect of *instruction type* indicated higher overall recognition accuracy in the positive than in the negative instruction category (F_1,49_ = 10.87; *p(F)* = 0.002; *η*_*p*_^*2*^ = 0.18). Furthermore, a significant main effect of *implementation status* indicated higher overall recognition accuracy on previously implemented compared to not implemented rules (F_1,49_ = 11.41; *p(F)* < 0.001, *η*_*p*_^*2*^ = 0.19) – this confirmed a trend from Experiment 1.

In contrast to Experiment 1 no significant interaction effect of *instruction type x implementation status* (F_1,49_ = 0.42; *p(F)* = 0.518; *η*_*p*_^*2*^ = 0.01) was observed. Positively instructed rules were recognized significantly better than negative ones if they had been implemented before (t(49) = 3.01; *p(t)* = 0.004) and if they had not been implemented before (t(49) = 2.65; *p(t)* = 0.011) – with the latter confirming a trend from Experiment 1. Previously implemented rules were recognized more accurately than not implemented ones in the positive condition (t(49) = 3.03; *p(t)* 0.004) with a trend-wise difference in the same direction being observed in the negative condition (t(49) = 1.85; *p(t)* 0.071).

#### Correlations between rule implementation and recognition

**Fig. 7:**
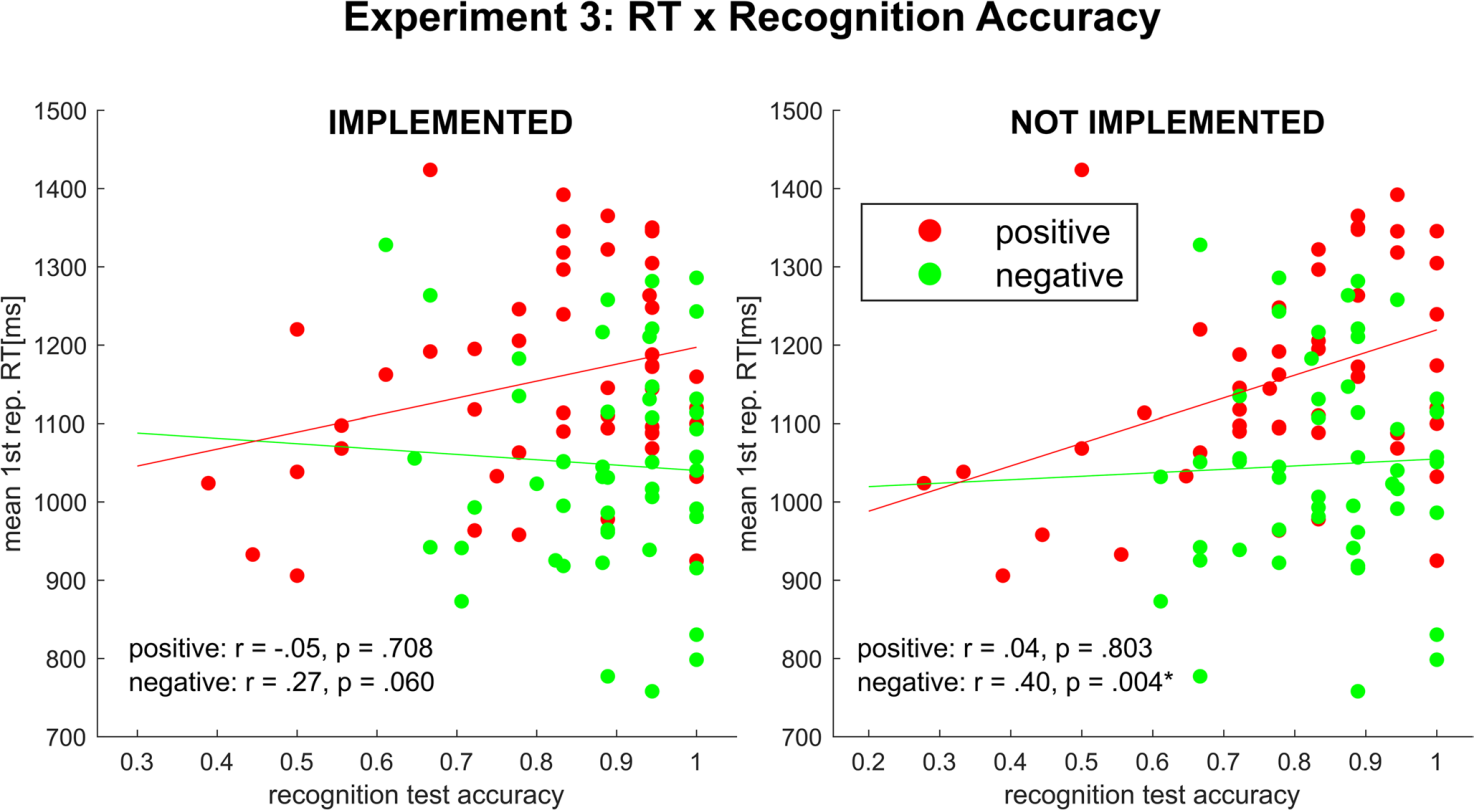
Scatter plots depicting the association between rule implementation (response time at first stimulus repetition) and recognition (accuracy) for implemented and not implemented rules per instruction condition in Experiment 3

In the positive condition, no significant correlation was observed between RT and recognition test accuracy (see Fig. 7), whereas a greater frequency of alternative-to-alternative (i.e., purely error-related) switches was associated with worse recognition test performance. This is in line with both, Experiment 1 and Experiment 2.

With respect to the negative instruction condition, more frequent alternative-to-alternative (i.e., purely correct) switches were significantly associated with better recognition test performance for both implemented and not implemented rules. A weak numerical but insignificant correlation into the same direction could be observed consistently for Experiment 1 but not for Experiment 2. At the same time, the correlation between recognition test accuracy on previously implemented rules and RT that was significant in Experiment 1 existed only numerically in Experiment 3. Furthermore, like in Experiment 1 and different from Experiment 2, recognition test accuracy for not implemented rules was significantly associated with higher implementation RT at all stimulus repetitions. For an overview, please see Table 3.

**Table 3:** Experiment 3 – correlation between implementation markers and recognition test accuracy. Asterisks indicate significant pearson correlation coefficients at *p* <.05. Abbreviations: REC = recognition Test; IMP = implementation phase; a2a = alternative-to-alternative; RT = response time; SR = stimulus repetition

		Negative				positive			
Variable 1	Variable 2	**SR1**	**SR2**	**SR3**	**SR4**	**SR1**	**SR2**	**SR3**	**SR4**
*REC: implemented*	*IMP: RT*	*r* =.27	*r* =.23	*r* =.20	*r* =.15	*r* = −.05	*r* = −.17	*r* = −.24	*r* = −.13
	*IMP: a2a-switches*	-	*r* =.49*	*r* =.50*	*r* =.45*	-	*r* = −.51*	*r* = −.56*	*r* = −.49*
*REC: instructed*	*IMP: RT*	*r* =.40*	*r* =.35*	*r* =.34*	*r* =.32*	*r* =.04	*r* = −.11	*r* = −.07	*r* = −.06
	*IMP: a2a-switches*	-	*r* =.38*	*r* =.47*	*r* =.40*	-	*r* = −.41*	*r* = −.32*	*r* = −.30*

### Discussion

Using a larger sample, we replicated the key findings of Experiment 1: Response time was significantly higher for negative than for positive instruction implementation and not modulated by stimulus repetition (i.e., practice) indicating a persistent impact of the instruction-related S-R rule identity. Furthermore, a pronounced decrease of alternative-to-alternative switches in the negative condition was observed again, indicating the gradual evolution of a distinct implementation-related S-R rule identity in parallel.

The significant and persistent positive correlation between alternative-to-alternative switches and recognition test accuracy in the negative condition suggests that an intact instruction-related S-R association is utilized to choose between the two alternative response options at the moment of implementation. This finding further substantiates a non-significant trend in the same direction in Experiment 1. Furthermore, the relationship between implementation RT and recognition test accuracy for not implemented rules supports the idea that reduced implementation efficiency in the negative instruction condition is tightly associated with integrity of the instruction-related S-R association (of which the recognition test for not instructed rules is the purest indicator as it should be least biased by action-oriented processes). Importantly, this association was not even present numerically in Experiment 2, possibly hinting at a fundamental representational difference of negatively instructed versus neutrally instructed (and only later negated) S-R rules.

Some of the modifications that were made due to the fMRI setting might have influenced overall accuracy rates. Specifically, inserting an ITI in between instruction screens might have allowed for greater encoding depth and thus resulted in generally higher (implementation and recognition test) accuracy. Moreover, the direct transition between phases (i.e., without a phase-specific announcement) is a likely cause for increasing accuracy rates after the first stimulus repetition. Nevertheless, despite this level difference potentially induced by the fMRI-setup, the main results pattern was conserved, a pattern that is consistent with previous study results (Koch et al., 2003).

Overall, Experiment 3 confirms the most relevant results of Experiment 1. Consequently, this suggests that our results could not merely be attributed to the specific color assignment to instruction type, perceptual characteristics of the recognition test or the structure of the stimulus sequence (i.e., including direct repetitions or not).

## General discussion

### Summary

There are two major takeaways from this behavioral study in which we analyzed implementation and recognition test behavior for positively and negatively (or neutrally) instructed rules. First, in Experiment 1 we presented evidence that following negative instructions, behavior is determined by the interplay of two diverging S-R associations involving the same stimulus. While the (negated) instruction-related S-R association seems to be present throughout the entire learning block, there is an evolving (affirmative) implementation-related association that becomes increasingly distinct over the course of several implementation trials. These results were replicated in a larger sample in Experiment 3. This dual nature is generally in line with findings from negation research(Dudschig & Kaup, 2018) and generalizes them to a rapid IBL-context. Second, the comparison between experiments 1 and 2 revealed the benefit of an advance negative instruction over the initial encoding of a neutral S-R association that is negated only later, once rule execution is required. This benefit of advance negative instructions – possibly comparable to general instruction benefits over other kinds of learning(Fregni et al., 2024; Ruge et al., 2018) – suggests that they trigger early preparation processes as well. Importantly, actual implementation seems to benefit from preparation whereas later recognition is harmed. At last and although the main focus in this study was on characterizing negative instructions in IBL, it also clearly emphasizes the advantage of direct, positive instructions over instructions presented in different ‘indirect’ formats (Pereg & Meiran, 2021).

### Interplay and competition of related S-R associations

One of the key aspects in this study is the interplay of different S-R associations rooting in one (instructed) rule in the service of successful execution. This is reminiscent of findings from one of our previous studies(Baumann et al., 2023) in which we examined the impact of instructional load in ‘standard’ positive instructions. Specifically, at high instructional load, the implemented response differed from the instructed response in a considerable proportion of trials. Therefore, a rule representation related to the instruction and a self-generated rule representation related to (previous) implementation – both involving the same stimulus but different responses – could potentially compete at the upcoming stimulus repetition instance. While subjects tended to generally stick to the previously implemented response (i.e., relying on the implementation-related S-R), the instruction-related S-R association seemed to have a persistent impact on rule execution in such conflict trials (Baumann et al., 2023). Thus, the instruction-related S-R association seemed to be continuously represented in some format despite the obvious incapability to actively retrieve it in the first place – resulting in objectively erroneous responding at some point during implementation. The results of the current study suggest that for negative instructions, however, the existence of two entities involving the same stimulus but being associated with different responses is a by-product of if not a prerequisite for correct behavior. Hence, negative instructions might indeed enable the investigation of instruction-induced conflicts at the rule-identity level without having to rely on erroneous behavior as a mediator.

This also bears relevance with respect to studies in which IBL was compared to trial-and-error learning (Fregni et al., 2024; Ruge et al., 2018). Here, it was found that even when considering only trials after which a rule had been successfully inferred from trial-and-error learning an advantage of rules learnt via instruction persisted. This implies that previous (incorrect) implementation attempts have a lasting adverse effect on rule execution – even if the erroneous implementation was merely observed (Fregni et al., 2024). The current study results are in line with these findings in so far as they – like Baumann et al. (2023) – emphasize that even an S-R association that is not related to implementation at all (i.e., merely instructed or not implemented) contributes to a lack of behavioral efficiency. At the same time, while it is relatively clear that this lack of efficiency is associated with detrimental influences of at least two competing S-R associations in the aforementioned studies (Baumann et al., 2023; Fregni et al., 2024; Ruge et al., 2018) the second, not to be implemented S-R association (i.e., instruction-related) might be supportive of successful rule implementation to some extent. A qualitative difference like this mostly becomes evident in the sustained implementation costs associated with negative instructions in the current study and negated rules more generally (Dudschig & Kaup, 2018; Wirth et al., 2023). As elaborated on above, these costs might originate partly from making active use of the instruction-related S-R association in order to generate a response (i.e., ‘negation cost’ and ‘choice cost’) and partly from it interfering with the evolving implementation-related S-R association (i.e., ‘interference cost’). It remains subject to further studies to quantify the relative influence of each cost type at each stage of implementation.

### Instruction-related/implementation-related vs. declarative/procedural

In this study we have used the terms *instruction-related* and *implementation-related* to characterize the S-R association types that are primarily associated with the respective stages in a learning block. While one might argue that a single implementation does not equal the formation of an effective S-R association, the repeated reliance on the very same response to a given stimulus (as reflected by significantly decreasing switching rates) that in addition adversely affects recognition test performance can be taken as evidence for the formation of exactly such an association. As elaborated on above, in the case of negative instruction following, this distinction can be objectivized by the identity that the S-R associations related to both respective stages bear. When considering the positive instruction condition it is well possible that there is also a distinction between the S-R association given by the instruction and that shaped through implementation. In a behavioral study, for example, Monsell and Graham(2021) utilized phonological properties of their stimulus material to draw conclusions about the format of the S-R association guiding implementation at a certain point. To characterize the two states between which they differentiated, they used the terms ‘declarative’ and ‘procedural’ with the former referring to the initial, abstract format susceptible to phonological interference and the latter referring to a more concrete, partly automatized(Mohr et al., 2016) format robust to such kind of interference. This seems to parallel some of the aspects that we intended to capture by the distinction made in the current study with ‘instruction-related’ corresponding to ‘declarative’ and ‘implementation-related’ corresponding to ‘procedural’. One question that remains is to which degree both of these distinctions actually overlap. This is particularly interesting as the declarative/procedural distinction does not only refer to the repeated implementation(Monsell & Graham, 2021; Ruge & Wolfensteller, 2010) or instruction(Pfeuffer et al., 2018; Ruge et al., 2024) but is also highly relevant already at the very core of each S-R transfer from instruction to implementation. Precisely, as mentioned in the introduction, it is commonly assumed that the declarative format of an S-R association is not sufficient for implementation but that its translation into an action-oriented procedural format is required (Brass et al., 2017). Taking this for granted, after (first time) implementation the existence of a procedural S-R representation is definitive. Moreover, research on ‘automatic effects of instructions’(Liefooghe et al., 2012; Meiran et al., 2015) suggests that such a procedural format already exists even before overt implementation, triggering exactly these effects. This makes it somewhat difficult, to strictly relate the declarative and procedural format of one and the same (positive) S-R rule to a precise point in time – as the continued presence of the abstract format of the rule is likely (Formica et al., 2020a; González-García et al., 2020b). Consequently, it is complicated to distinguish between both. The instruction-/implementation-related conceptualization, however, assigns the S-R representations to the phases they (first) overtly appear in and could thus be thought of as some kind of virtual ‘timestamp’. Importantly, it does not rule out the possibility that there exists a procedural representation of the instruction-related S-R association and that the implementation-related representation becomes represented declaratively as well. Nevertheless, assuming that a procedural representation is an essential requirement for implementation and that this representation can only include one response at a time and is thus inherently concrete(Brass et al., 2017; Ruge et al., 2024) we could conclude that, in general, a predominantly procedural representation should underlie the implemented rule. The sum of rule implementation instances – and the underlying procedural format – can be tracked in the form of the manifesting implementation-related S-R association. The instruction-related S-R association, in turn, that is never actually implemented should predominantly exist in a declarative format – especially when relevant for later recognition. Thus, the instruction- and implementation-related conceptualization might indeed be useful as a proxy to approach the declarative/procedural distinction. In this regard the negative instruction condition might allow for a more objective dissociation as it requires fewer assumptions about the properties of the two latent states but could be accomplished by the two distinct S-R identities. It remains subject to further studies to assess whether neural signatures of instruction-/implementation-related S-R associations resemble those of procedural/declarative representations (Cole et al., 2016; Formica et al., 2020b, 2022; González-García et al., 2020b; Muhle-Karbe et al., 2017; Ruge et al., 2019; Ruge & Wolfensteller, 2010).

### Informative vs. neutral instructions

Another interesting aspect of the current study is that it is informative with respect to the very basic character of instructions. The experiment comparison revealed differences between both experiments with respect to rule implementation as well as to the recognition test. Importantly, these effects are rooted in the way stimulus-position associations are presented during the initial instruction phase. The fundamental difference between both experiments is that informative instructions from Experiment 1 (and Experiment 3) allow for inference on relevant future behavior whereas neutral instructions from Experiment 2 do not. This is reminiscent of studies in which instructed S-R associations (to be implemented) were compared against ‘instructed’ object-color (OC) associations that were to be recognized later on (Bourguignon et al., 2018; Hartstra et al., 2011). Similar to the neutral instructions used in our Experiment 2 the OC associations did not allow for a particular response to be prepared. Relatedly, other studies(Formica et al., 2020a; González-García et al., 2020a, b) have used a paradigm in which several S-R rules are presented initially and only become activated subsequently by means of a retro-cue. This was done in order to localize the point in time at which the transformation from declarative to procedural rule state happens (see above). However, different from these approaches, our Experiment 2 included an association between stimulus and response which needed to be learnt but lacked information on *how* it was to be used in the service of successful rule implementation. In other words, although being highly relevant for behavior – a correct response cannot be provided without the instruction-related S-R association in mind – it is equally probable that the associated response key is to be pressed (i.e., affirmative cue), or that another key is to be pressed (i.e., negation cue), in the service of goal-directed action. While it remains debatable whether this neutral instruction condition had indeed resulted in any action tendency to be precluded (see above), it, nonetheless offers an additional perspective on what actually makes up the ‘abstract’(Brass et al., 2017) or ‘symbolic’ (Ruge & Wolfensteller, 2010) component in instruction-based learning: Is it a representation that resembles the ‘neutral’ rule from Experiment 2 which encompasses stimulus and response information but is completely void of information about any correct action tendency? Or does it contain information about the appropriateness of the action as well – as was the case in Experiment 1 and Experiment 3 – and simply is not yet bound to execution? At the very basic level it could speak to the question of whether the mere presence of an attended stimulus-response link is sufficient to trigger a future action tendency or whether an actual proactive preparation(Cole et al., 2017) to future implementation is a requirement. Importantly, this is only possible in the current setup as the possibility of negative and positive rules enables the initial encoding ambiguity.

### Limitations and future directions

In the current study, we have assessed behavioral characteristics following negative instructions in the context of novel, rapidly learned S-R associations. Two aspects in particular should be considered when interpreting our results.

First, by their very nature, negative instructions allowed us to examine the interplay of instruction-related and implementation-related rule representations as a potential means to dissociate functional rule states in IBL. In order to obtain a valid estimation of instruction-related representational integrity, the inclusion of the recognition test was necessary. As this test was placed after actual implementation and test performance was incentivized, it could have influenced implementation behavior. Precisely, maintenance of the instruction-related S-R association might have obstructed rule transformation processes. This aspect, is clearly relevant, as even for positive instructions reports exist in which mere subsequent recognition of an instructed rule did (Liefooghe & Houwer, 2018) or did not (Liefooghe et al., 2012) impact rule transformation processes. With respect to the current study, Experiment 2 represents a ‘lower boundary’ of what is to be expected once the instruction-related S-R association is maximally weighted regarding the degree to which it influences implementation. In comparison, once negation information was available before implementation (Experiment 1 and 3), we observed a clear trend towards advance proceduralization (reflected in implementation RT and switching rates) at the cost of instruction-related S-R association integrity (reflected in recognition test accuracy). Nevertheless, it remains subject to further studies to assess whether the behavioral patterns of Experiment 1 and 3 could be generalized to a setup in which subsequent rule recognition does only play a minor role or none at all.

Second, like previous studies examining positive instructions (e.g., Palenciano et al., 2024; Ruge et al., 2019), we used an experimental setup that involved multiple (here: three) response options. As mentioned above, this comes at an imbalance in the number of valid response options between positive and negative instructions in the implementation phase, affecting implementation accuracy, response switching and likely also RT (by inducing a ‘choice cost’ component). However, after weighing several alternative design options, all things considered, we decided for the current experimental setup despite some potential limitations. One important reason was that only the three-response setup enabled tracking the dynamics of alternative-to-alternative response switches. This analysis type would not be possible in an alternative setup with only two response options (i.e., one correct and one incorrect option per rule) as was employed by most of the studies examining either negation or indirect instruction effects (e.g., Dudschig & Kaup, 2018; Liefooghe et al., 2016; Pereg & Meiran, 2021). Moreover, such a two-response setup might come with unwanted short-cut strategies. Specifically, all S-R associations can in principle be memorized by linking each stimulus to only one response option with this either being executed or negated subsequently (as the only other option would be the correct one then). Not only would such a strategy place a heavy confound on the distinction between negative and positive instructions in the current experiment, but subjects would also be increasingly motivated to encode all stimuli in relation to each other instead of individual S-R rules. Another alternative design option would be to introduce response ambiguity in the negative condition and positive condition alike (i.e., two correct response options per rule). For example, this could be achieved by presenting two explicit positive instructions involving the same stimulus (e.g., for the word stimulus ‘coffee’, both a middle and ring finger response would be correct). This setup, however, would induce its own interpretational problems due to perceptual differences during instruction presentation and an imbalance with respect to instructional load. Moreover, the present study was based on matching positive and negative instruction types with respect to unambiguity at the level of instruction-related S-R associations. This matching would be counteracted by such a setup with asymmetry being deferred to the recognition test. To conclude, these approaches in order to disentangle cost components are appealing but also have inherent shortcomings. Therefore, this matter requires and deserves a detailed examination of its own which is clearly beyond the scope of the current study. Meanwhile our results should not be considered a definitive quantification of pure negation versus affirmation effects but much rather an initial step towards a characterization of negative instructions in IBL for which positive instructions serve as a reference or baseline. For that purpose, the current design captures both instruction types within their ‘naturalistic’ setting, that is, the positive instruction specifies the one response (out of several options) that is to be executed whereas the negative instruction is more open in that it only specifies which action is not be executed - inherently leaving multiple permissible alternatives (cf. Proctor & Xiong, 2017).

## Conclusion

Our study tracked rule implementation and recognition following novel, negative instructions. Implementation efficiency clearly benefitted from negation information being available prior to first time overt rule execution. This suggests that the negation became proactively integrated into the current task model. However, the resulting rule transformation processes were clearly not as advanced as those observed for standard positive instructions. Furthermore, our results emphasize the coexistence and interplay of distinct instruction-related and implementation-related rule representations as a unique property of negative instructions. While the instruction-related S-R association exerts a persisting influence throughout repeated rule implementation, an implementation-related representation becomes increasingly distinct. Necessarily these two representations differ objectively at the level of rule (that is, stimulus-response association) identity. Thus, negative instructions potentially offer a novel framework to dissociate rule representation levels and quantify proactive preparation during learning. Future research should explore these mechanisms further, incorporating both behavioral and neural approaches to deepen our understanding of instruction-based learning.

## Supplementary Information

Below is the link to the electronic supplementary material.

Supplementary Material 1 (DOCX 267 KB)

## Data Availability

The raw behavioral data for all experiments are available at https://osf.io/839dk/with the DOI 10.17605/OSF.IO/839DK being assigned. Further experimental materials can be made available upon request.

## Footnotes

1 Please note, that in the negative condition the first type (instructed-to-alternative) represents a switch from the incorrect to a correct response and the second type (alternative-to-instructed) represents a switch from the correct to an incorrect response. In the positive condition the interpretation is exactly the opposite.
