# Supplementary material for: Implementation and recognition of novel negatively instructed stimulus-response rules
Source: Psychol Res. 2025 Nov 29;90(1):3. doi: 10.1007/s00426-025-02212-2 (PMC12664855; doi:10.1007/s00426-025-02212-2)
Supplement: Supplementary file 1 — Supplementary Material 1 (DOCX 267 KB) [file 426_2025_2212_MOESM1_ESM.docx]

Supplementary Materials

1. General Instruction Experiment 1

Participants were provided with general task instructions before the start of the experiment (i.e., before the training blocks). As part of the computerized experiments, task instructions were presented on multiple screens of which the most critical to the understanding of the key condition difference (i.e., instruction type: positive vs. negative) are printed below. The original German text (italic) and an English translation (standard font) are provided.

1.1 General Instruction: paradigm outline

*Entscheidend für Phase 2 sind Position und Farbe eines Wortes in Phase 1.*

*Wird ein Wort in Phase 1 in grüner Farbe angezeigt, soll in Phase 2 angegeben werden, wo sich dieses Wort in Relation zur '||'-Begrenzung in Phase 1 befunden hat.*

*Wird ein Wort in Phase 1 in roter Farbe angezeigt, soll in Phase 2 angegeben werden, wo sich dieses Wort in Relation zur '||'-Begrenzung in Phase 1 NICHT befunden hat.*

*WICHTIG: Alle Worte werden in Phase 2 in derselben Farbe angezeigt wie in Phase 1! Nicht jedes Wort aus Phase 1 muss zwangsläufig in Phase 2 vorkommen.*

The position and color of a word in phase 1 are critical for phase 2.

If a word is displayed in green in phase 1, you should indicate where this word was located in relation to the “||” boundary in phase 1.

If a word is displayed in red in phase 1, you should indicate where this word was NOT located in relation to the “||” boundary in phase 1.

IMPORTANT: In phase 2, all words are displayed in the same color as in phase 1! Not every word from phase 1 must necessarily re-appear in phase 2.

1.2 General Instruction: positive condition

*Im Beispiel auf der linken Seite sehen Sie ein in grüner Farbe angezeigtes Wort (Phase 1). Hier sollte in Phase 2 mit einem Tastendruck auf die Taste reagiert werden, die der Position des angezeigten Wortes entspricht.*

*In diesem Fall steht das angezeigte Wort in Phase 1 näher zur linken '|'-Begrenzung. In Phase 2 sollte auf dasselbe Wort, ohne '||'-Begrenzungen, also mit einem Tastendruck auf die Taste 'T' geantwortet werden.*

In the example [see supplementary Figure 1a], you can see a word displayed in green (Phase 1). In Phase 2, you should respond by pressing the key that corresponds to the position of the displayed word.

In this case, the word displayed in Phase 1 is closer to the left “|” boundary. Therefore, in Phase 2, the same word – without “||” boundaries – should be responded to by pressing the “T” key.

1.3 General Instruction: negative condition

*Im Beispiel auf der linken Seite sehen Sie ein in roter Farbe angezeigtes Wort (Phase 1). Hier sollte in Phase 2 mit einem Tastendruck auf eine der Tasten reagiert werden, die der Position des angezeigten Wortes NICHT entsprechen.*

*In diesem Fall steht das angezeigte Wort in Phase 1 näher zur rechten '|'-Begrenzung. In Phase 2 sollte auf dasselbe Wort, ohne '||'-Begrenzungen, also mit einem Tastendruck auf eine der Tasten 'T' oder 'Z' geantwortet werden.*

In the example [see supplementary Figure 1b], you can see a word displayed in red (Phase 1). In Phase 2, you should respond by pressing the key that does NOT correspond to the position of the displayed word.

In this case, the word displayed in Phase 1 is closer to the right “|” boundary. Therefore, in Phase 2, the same word – without “||” boundaries – should be responded to by either pressing the “T” or the “Z” key.


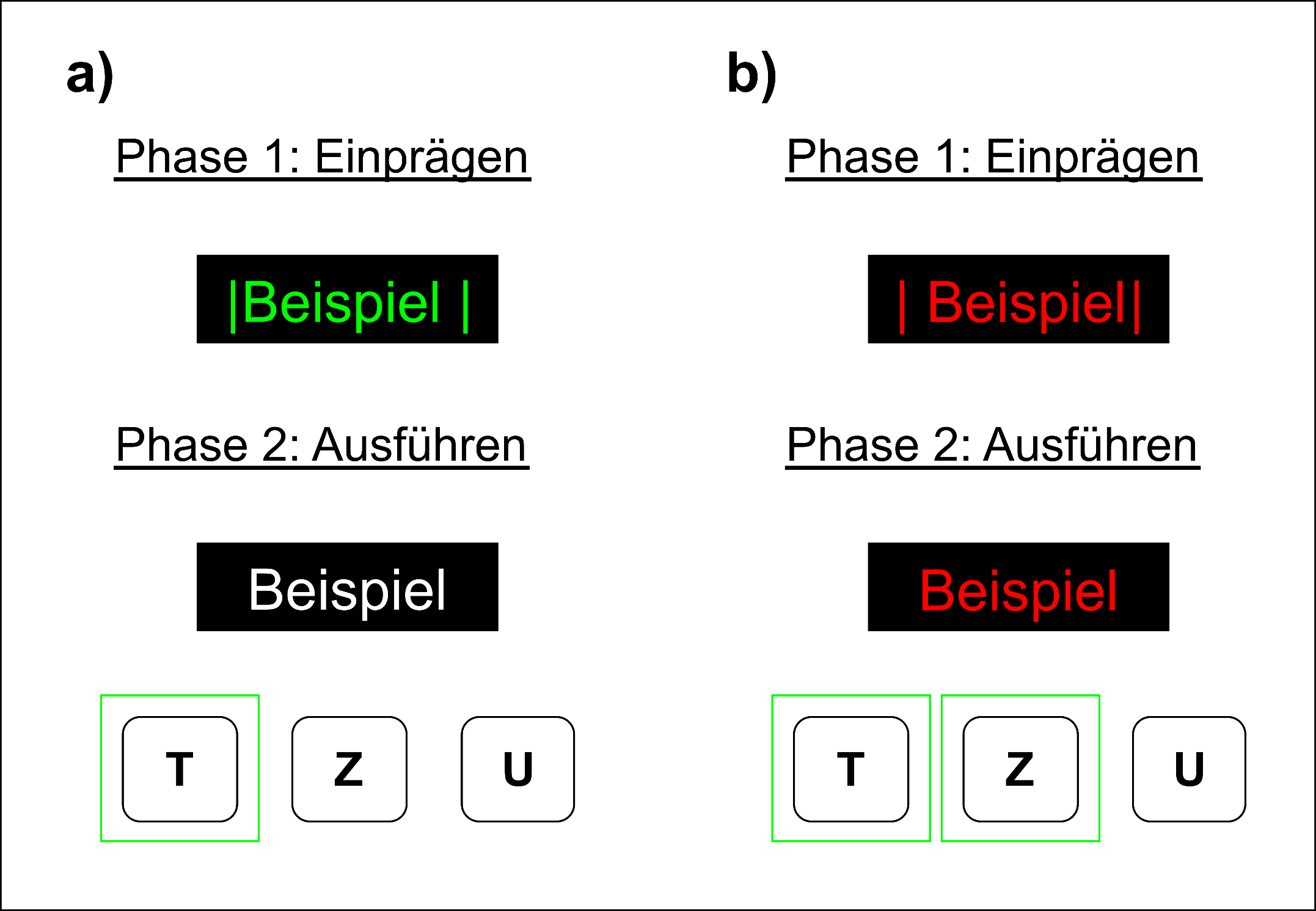


**Supplementary Figure 1.** Schematic example of the task phase structure that was shown to participants alongside general instructions for the positive (a) and the negative (b) condition.

2 All Experiments: Alternative switching definition

As described in the main text, we conducted a one-way repeated measures ANOVA – with the single factor *stimulus repetition* – per Experiment. No *instruction type* factor was included as, per definition, the proportion of switches amongst all correct-to-correct repetition sequences would be at zero in the positive condition. Thus, only the negative instruction condition is considered. Here the results corroborate the findings reported in the main text.

Specifically, across all experiments, the proportion of (alternative-to-alternative) switches amongst correct-to-correct sequences was significantly greater than zero as defined by the constant term across all stimulus repetitions (**E1**: F_1,29_ = 87.64, p(F) < 0.001, *η_p_^2^* = 0.75; **E2**: F_1,29_ = 291.76, p(F) < 0.001, *η_p_^2^* = 0.91; **E3**: F_1,49_ = 144.07, p(F) < 0.001, *η_p_^2^* = 0.75).

Most importantly, this proportion decreased across stimulus repetitions (**E1**: F_1.64,47.54_ = 4.41, p(F) = 0.024, *η_p_^2^* = 0.13; linear contrast: F_1,29_ = 9.29, p(F) = 0.005, *η_p_^2^* = 0.24; **E2**: F_1.57,45.62_ = 7.10, p(F) = 0.004, *η_p_^2^* = 0.20; linear contrast: F_1,29_ = 9.34, p(F) = 0.005, *η_p_^2^* = 0.24; **E3**: F_1.74,85.08_ = 18.22, p(F) < 0.001, *η_p_^2^* = 0.27; linear contrast: F_1,49_ = 28.86, p(F) < 0.001, *η_p_^2^* = 0.37). For an illustration, please see Supplementary Figure 2.

**
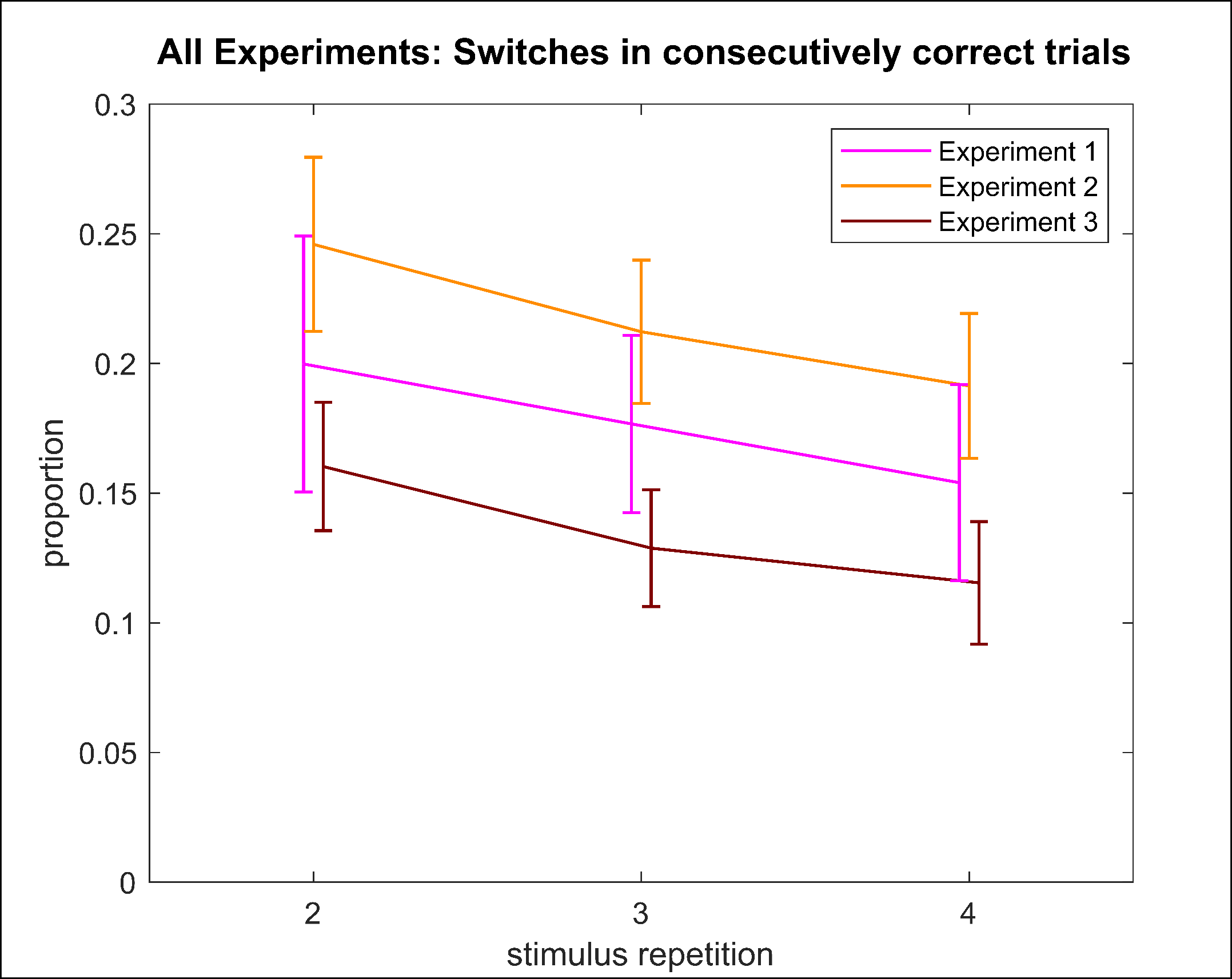
**

**Supplementary Figure 2.** Depiction of switching rate defined as the proportion of switches in consecutively correct (i.e., correct-to-correct) stimulus repetition sequences for all three Experiments. Per definition, this only refers to the negative condition.

3 Experiment 1: Experimental Stage RTs

In a follow-up exploratory analysis, we sought to test whether the difference between both instruction types was robust across the experiment. Therefore, the experiment was divided into two halves, resulting in an early (blocks 1 to 18) and a late (19-36) *experimental stage*, which was included as a factor into a 2-by-2-by-4 repeated measures ANOVA. The significant main effect of *experimental stage* (F_1,29_ = 38.74; *p(F)* < .001; *η_p_^2^* = 0.57) indicated higher overall RTs in the early compared to the late stage and a significant interaction of *instruction type* x *experimental stage* (F_1,29_ = 4.92; *p(F)* = .035; *η_p_^2^* = 0.15) indicating a more pronounced difference between instruction types (negative longer than positive) in the early as compared to the late experimental phase. None of the other interactions reached statistical significance (all F < 1.68; *p(F)* > .178; *η_p_^2^* < 0.06, linear contrast: all F < 1.72; *p(F)* > .199; *η_p_^2^* < 0.06).

Separately conducted ANOVAs for both experimental stages revealed a significant main effect of *instruction type* in the early (F_1,29_ = 58.35; *p(F)* < .001; *η_p_^2^* = 0.67) as well as in the late (F_1,29_ = 34.05; *p(F)* < .001; *η_p_^2^* = 0.54) experimental stage. This indicated a strong and sustained difference between both instruction types with RTs to negatively instructed rules being consistently slower than RTs to positively instructed rules.
